# PROTAC Enabling Formulation *In Vivo*: Implications of the Polymeric Carrier Eudragit E PO

**DOI:** 10.1021/acs.molpharmaceut.5c00303

**Published:** 2025-08-24

**Authors:** Nicole Hofmann, Florian Johann, Katharina Krollik, Andreas Marx, Heide Marika Duevel, Marc Lecomte, Meike Harms, Karsten Mäder

**Affiliations:** a Global Drug Product Development, Orals Development, the Healthcare Business of Merck KGaA, Frankfurter Straße 250, Darmstadt 64293, Germany; b Institute of Pharmacy, Faculty I of Natural Sciences, 9176 Martin Luther University Halle-Wittenberg, Kurt-Mothes-Strasse 3, Halle (Saale) 06120, Germany; c Site Management Lab Services, 2792 Merck KGaA, Frankfurter Straße 250, Darmstadt 64293, Germany; d Global Research and Development, NCE DMPK, the Healthcare Business of Merck KGaA, Frankfurter Straße 250, Darmstadt 64293, Germany

**Keywords:** proteolysis targeting chimeras, amorphous solid dispersions, enabling formulation, Eudragit, bile salts, bioavailability

## Abstract

Proteolysis targeting chimeras (PROTACs) are heterobifunctional degraders with a unique mode of action that permits access to “undruggable” targets. These molecules pose challenges in terms of solubility and bioavailability due to their physicochemical properties. So far, very little information is available on the potential of enabling formulations of PROTACs in pharmacokinetic studies. In a previous work of our group, amorphous spray-dried formulations (SDDs) of the model PROTAC MS4078 were developed. However, the potential of the amorphous solid dispersions of the PROTAC MS4078 has not yet been examined *in vivo*. The present study reports on the evaluation of an SDD containing MS4078 and E PO in comparison to a solution vehicle in a pharmacokinetic study in mice. Unexpectedly, very little exposure was found for both formulations. For a deeper investigation of their supersaturation and precipitation performance, the two formulations were tested in a two-stage precipitation assay with media that mimic physiological conditions in mice, which includes various bile salt and phospholipid concentrations. In this assay, the solution vehicle turned out to be a potent solubility enhancer. For the SDD, however, very different and complex dissolution profiles were obtained. The concentration at each time point was dependent on the bile salt and phospholipid concentration. Further *in vitro* tests revealed that E PO, the bile salt, and phospholipid interacted and induced a phase separation. This affected the solubilization and stabilization of the model PROTAC and may explain the differences regarding its *in vitro* performance. These findings are important to consider when designing future studies including E PO as polymeric carrier and species with high bile salt and phospholipid concentrations.

## Graphical abstract

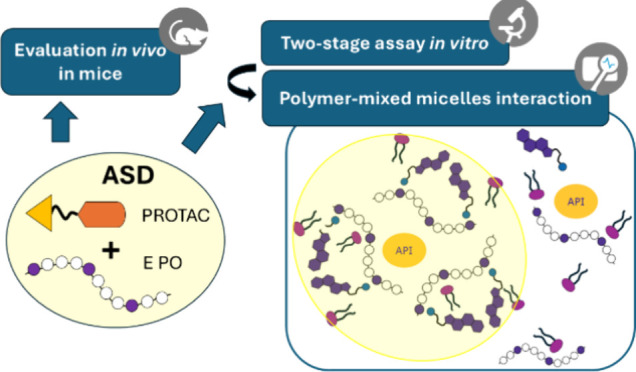

## 1 Introduction

Targeted protein degraders, also known as PROTACs, a trademark coined by the US company Arvinas, describe a group of molecules that are designed to bind to an E3 ligase, a component of the proteasome-ubiquitin system, and, simultaneously, to a protein of interest. Due to the induced proximity between the ligase and the protein, a transfer of ubiquitin onto the protein of interest is enabled, which marks it for degradation.^1,2^ Because of the two functional parts of a PROTAC – each about the size of a small molecule on its own – the physicochemical properties in most cases pose a challenge in terms of solubility, lipophilicity, permeability, and consequently, oral bioavailability.^3,4^ Thus, enabling formulations are likely needed for the development of oral formulations for these degraders. Despite this need, there is very little information available on formulation attempts and pharmacokinetic (PK) studies so far. In particular, there is hardly any data on pharmacokinetic studies including amorphous solid dispersions of PROTACs.^5,6^ In a previous publication by our group, ASDs of a model PROTAC, MS4078, were developed and evaluated in terms of their feasibility for this modality.^7^ MS4078 (structure see Figure S1) is a Cereblon-recruiting degrader of the anaplastic lymphoma kinase developed as a treatment for non-small cell lung cancers.^8^ The compound was formulated in amorphous spray-dried formulations containing Eudragit E PO (E PO) or Soluplus as polymer. In both formulations, a high supersaturation was achieved in a single-stage dissolution assay in FaSSIF (fasted state simulated intestinal fluid). Relative to the neat, amorphous API, both spray-dried formulations provided a more than 70fold supersaturation.

E PO, which was used in the study as polymeric matrix, is not only a carrier in ASD formulations,^9^ but also a broadly used excipient in pharmaceutical coatings. It was developed for taste and odor masking, as well as protection of the coated material from light and moisture.^10^ E PO is a copolymer, with N,N-dimethylaminoethyl methacrylate, methyl methacrylate, and butyl methacrylate derived side chains (structure see Figure S1). The solubility of the polymer is strongly pH-dependent due to the presence of amine groups. In media with pH values above 5, the polymer is not dissolved, but swellable and permeable. At pH values below 5, the amine is protonated, and the polymer will be dissolved. As an ionizable polymer, E PO is prone to interact with negatively charged molecules like active pharmaceutical ingredients (APIs)^9,11^ but also bile salts, which has been investigated in several studies. It has been shown that these interactions may influence the exposure *in vivo*. For example, Saal et al. observed a delayed release profile and influence on pharmacokinetic descriptors (*c*_max_, *t*_max_, AUC) after administration of a solution vehicle containing un-ionized compounds and E PO as a solubility enhancer.^12^ The authors hypothesized that the pH shift upon passage to the rats’ intestines led to precipitation of both the drug and the polymer. In a different study, Schlauersbach et al. investigated the combination of bile salt interacting drugs with several polymers, including E PO.^13^ The structure of FaSSIF micelles changed in the presence of the polymer due to interactions that interfered with the interplay of bile salts and the compounds. They hypothesized that the interaction might have an impact on the solubilization of bile salt interacting drugs. These publications indicate that EPO-bile salt interactions are of high relevance *in vitro* and *in vivo.*

Bile salts and phospholipids emulsify hydrophobic and lipophilic substances like pharmaceutical compounds in mixed micelles in the intestine and are crucial for their uptake.^14,15^ Therefore, their implementation in biorelevant media is important for a more accurate representation of the behavior of drugs and formulations in the gastrointestinal tract. In pursuit of the best equivalent to human intestinal fluid (HIF) for *in vitro* assays, FaSSIF-V1, a mixture of sodium taurocholate (TC) and phosphatidylcholine (PC) (structures see Figure S1) was developed in 1998.^16^ These components form colloidal structures (micelles, uni- and multilamellar vesicles of different morphologies) as equivalent to the mixed micelles in the human intestine.^17^ Since then, further types have been developed, where the composition was adapted to the latest data about HIF (FaSSIF-V2, FaSSIF-V3), whereas FaSSIF-V1 remained the most popular and accepted version. Besides, adapted media for different animal species like rats have been developed^18^ and an improved *in vivo-in vitro*-correlation (IVIVC) has been demonstrated.^19,20^ Nevertheless, mice have not been characterized to the same extent, which is a huge gap since an increasing number of early PK studies are conducted in this species.^21^

The accurate representation of pH conditions in the intestine is another important factor in biorelevant dissolution and precipitation assays. There are numerous examples of pharmaceutical APIs that are weakly basic drugs like ketoconazole, ritonavir, dipyridamole, and the model PROTAC MS4078. These molecules are prone to precipitation because of pH shifts during the gastrointestinal passage.^22^ Therefore, biorelevant assays that comprise two stages, one with a low pH, simulating the stomach, and a second one with a higher pH, the “intestine”, are especially valuable.

The present study reports on the evaluation of a first PK study in mice of the amorphous spray-dried dispersion (SDD) containing MS4078 and E PO^7^ and the comparison to a solution vehicle (5% DMSO, 20% Kolliphor HS15 in water). For a more detailed investigation of the formulations, they were additionally tested in a two-stage precipitation assay simulating the conditions in mice. FaSSIF was prepared using a buffer system with a pH of 5 and for a more comprehensive picture, a broad range of taurocholate/phosphatidylcholine (TC/PC) concentrations was employed. In the second part, the implications of E PO as a polymeric carrier were evaluated to gain more information on the dissolution, stabilization, and precipitation of MS4078 from SDD formulations. The findings were finally compared to the *in vivo* results.

## 2 Materials and Methods

### 2.1 Materials

The model PROTAC MS4078 was obtained from MedChemExpress (Monmouth Junction, NJ, USA). Eudragit E PO (E PO) was provided by Evonik (Darmstadt, Germany), 3F Powder (containing bile salts (sodium taurocholate (TC)) and lecithin (phosphatidylcholine (PC)) for the preparation of FaSSIF-V1 was purchased from Biorelevant (London, UK) and Glafenine was obtained from Merck (Darmstadt, Germany). Hypergrade acetonitrile (ACN) and methanol (MeOH), analytical grade trifluoroacetic acid (TFA), dichloromethane (DCM), dimethyl sulfoxide (DMSO), Polysorbate 20 (Tween 20), sodium hydroxide (NaOH), sodium chloride (NaCl), sodium dihydrogen phosphate (NaH_2_PO_4_), 1 N hydrochloric acid (HCl), 1 N sodium hydroxide solution (NaOH), deuterium oxide (D_2_O), and citric acid monohydrate were produced by Merck (Darmstadt, Germany). Methocel K4M was obtained from Colorcon (Kent, UK), Kolliphor HS 15 was provided by BASF (Ludwigshafen, Germany). All aqueous solutions were prepared with purified water (Milli-Q, Merck, Darmstadt, Germany).

### 2.2 Preparation of Biorelevant Media

#### 2.2.1 Preparation of Simulated Gastric Fluid (SGF)

SGF pH 4 was prepared by weighing in 1000 mg of NaCl and adding 500 mL Milli-Q water. The pH of the solution was adjusted to 4 ± 0.05 using 1 N HCl (780 pH meter, Metrohm, Filderstadt, Germany).

#### 2.2.2 Preparation of Buffer and cFaSSIF Types

A citrate-phosphate buffer was prepared for the citrate-based FaSSIF types (cFaSSIF) (Table S1). For a detailed discussion on the choice of buffer type and pH, please refer to Section 4. NaCl, NaOH, NaH_2_PO_4_ and citric acid monohydrate were dissolved in Milli-Q water, and the pH was adjusted to 5 ± 0.05. The cFaSSIF types were prepared by weighing the corresponding amount of 3F Powder into a snap cap vial and adding citrate-phosphate buffer pH 5 (see Table 1). The solution was stirred for 2 h before further use. Please note that taurocholate and phosphatidylcholine (in the following TC/PC) are constituents of 3F Powder. By applying different amounts of 3F Powder, several concentrations were prepared for this study which will be abbreviated by c_TC/PC_ or cFaSSIF with an index indicating the concentration (see Table 1).

**Table 1:** Citrate-Based FaSSIF Types pH 5

Type	c_TC/PC_ (relative to FaSSIF-V1)	Bile salt (taurocholate, TC) [mM]	Phospholipids (phosphatidylcholine, PC) [mM]
cFaSSIF_1*x*_	1x	3	0.75
cFaSSIF_2*x*_	2x	6	1.5
cFaSSIF_5*x*_	5x	15	3.75
cFaSSIF_10x_	10x	30	7.5
cFaSSIF_20x_	20x	60	15
cFaSSIF_40x_	40x	120	30

### 2.3 Solubility of MS4078

The solubility of MS4078 was evaluated by weighing an excess amount of the compound into vials and adding a prewarmed buffer or cFaSSIF types (cFaSSIF_1*x*_, cFaSSIF_5__*x*_ *,* and cFaSSIF_10x_ ). The suspension was stirred at 37°C for 24 h at 300 rpm. Samples were withdrawn from the suspension, and centrifuged at 15000 rpm (Mikro 200 R, Hettich, Tuttlingen, Germany) and the supernatant was diluted 1:1 with ACN. The dissolved concentration was determined by ultraperformance liquid chromatography (UHPLC, see Section 2.7.1). The experiments were carried out in triplicate.

### 2.4 Manufacturing of Formulations

#### 2.4.1 MS4078 in Kolliphor Vehicle

The API in a Kolliphor HS15 solution vehicle for pharmacokinetic studies and precipitation assays was prepared by dissolving 20 mg/mL MS4078 in DMSO. Then, the API solution was slowly added to a mixture of Kolliphor HS15 in water under continuous stirring. The vehicle was protected from light and stirred overnight at 300 rpm to obtain a clear, yellow solution. The final concentrations were 1 mg/mL of MS4078, 5% (w/V) DMSO and 20% (w/V) Kolliphor HS15. The iv dosing solution was filtered before administration. Dosing solution concentrations: 1 mg/mL (po) and 0.1 mg/mL (iv), respectively.

#### 2.4.2 Spray-Dried Formulation

For the manufacturing of the spray-dried formulation containing 10% (w/w) MS4078 and 90% (w/w) E PO, the components were dissolved in dichloromethane/methanol (90:10 (V/V), 2% (w/w) solution), dispersed by airflow of 10 L/min, and dried at 70°C using the 4M8-TriX (ProCepT, Zele, Belgium). More details on manufacturing and analytics (content, purity, solid state and dissolution performance) can be found in a previous work of our group.^7^

#### 2.4.3 Dispersion of SDD Formulation in Citrate Vehicle

A dispersion of the SDD formulation was prepared for the pharmacokinetic studies and two-stage assays. Therefore, a 100 mM citrate buffer pH 3 containing 0.5% (w/V) Methocel K4M and 0.25% (w/V) Tween 20 was prepared by dissolving the corresponding amount of Methocel K4M, Tween 20 and citric acid monohydrate in Milli-Q water. The pH of the vehicle was adjusted to pH 3 ± 0.05 using 1 N NaOH solution. An equivalent of 10 mg/mL SDD powder was weighed into a snap cap vial, and the citrate vehicle was added under stirring at 300 rpm.

### 2.5 *In Vivo* Pharmacokinetic Studies in Mice

To evaluate the *in vivo* performance of the MS4078 SDD formulation, pharmacokinetic studies in mice were conducted and compared to the corresponding Kolliphor solution vehicle. The API in Kolliphor vehicle (see Section 2.5.2) and the SDD formulation ([Sec sec2.5.2]) were given in a single-dose study to female CD-1 mice (Charles River Laboratories, Sulzfeld, Germany). The animals were kept in group housing in conventional cages with elevated grids. Food and water were offered ad libitum to the animals.

All animal procedures were performed in accordance with the Guidelines for Care and Use of Laboratory Animals of Merck KGaA/EMD Serono and approved by the internal Animal Ethics Committee of Merck/EMD Serono. All used laboratories and CROs have been audited by the Merck/EMD Serono animal welfare office, and the animal work was approved by the local ethics committees responsible for the respective CROs/laboratories. For the study using the SDD formulation conducted at Nuvisan (Grafing, Germany), the respective unique study number was #22/312 and the animal testing license ROB-55.2–2532.Vet_02–21–25, approved by the government of Upper Bavaria (Regierung Oberbayern). For the Kolliphor vehicle studies, all parts of the study plan concerning animal care have been reviewed by the EMD Serono “Institutional Animal Care and Use Committee (IACUC)” which reports to the EMD Serono Billerica site Head. Protection of animals used, housing and welfare are guaranteed according to IACUC protocol 017–004 (Pharmacokinetic Studies in Rodents). The unique study identifier is BOS-PK-20–009.

#### 2.5.1 Kolliphor Vehicle

The study was carried out at EMD Serono (Billerica, MA, USA). A total of 21 mice (female, CD-1) were divided into groups of 12 and 9 animals. The solution vehicle was prepared according to Section 2.4.1. Doses of 0.5 mg/kg of MS4078 were administered intravenously (dose volume: 5 mL/kg) to 12 animals and blood samples (retro-orbital) were sequentially drawn from 3 animals per sampling point at 0.1, 0.25, 0.5, 1, 2, 6, and 24 h after dosing. 10 mg/kg were administered orally (dose volume: 10 mL/kg) to the second group of 9 animals. After dosing, blood samples from 3 animals per sampling point were sequentially drawn at the time points described above, except for the 0.1 h sampling point. The blood cells were dissevered by centrifugation (2000 g, 5 min). Plasma samples were extracted using a protein precipitation reagent (ACN, 4:1 (V/V)). After the addition of 50 μL methanol, the concentration was determined by LC-MS/MS (generic method, based on tuning data).

#### 2.5.2 SDD Formulation

Mouse PK studies of the enabling formulation were conducted at Nuvisan GmbH (Grafing, Germany). To determine oral bioavailability of MS4078/SDD, the SDD formulation (dose: 10 mg/kg MS4078) was weighed in, dispersed in a citrate vehicle (see Section 2.4.3), and administered orally (gavage, dose volume: 10 mL/kg) to three female mice (Crl:CD1­(ICR)). Blood samples were taken retro-orbitally 0.25, 0.5, 1, 2, 4, 6, and 24 h after oral administration. Plasma was obtained by centrifugation (10,000 g; 4 °C; 5 min) and stored at −20 °C until UHPLC-MS/MS analysis. By addition of 100 μL internal standard (Glafenine) solution in ACN, the proteins were precipitated and dissevered by centrifugation. 50 μL of supernatant were diluted 1:1 with a solution of 0.1% formic acid in water and the concentration of MS4078 was determined by UHPLC-MS/MS (generic method, based on tuning data).

#### 2.5.3 Pharmacokinetic Data Analysis

Plasma concentrations were determined for each time point sampled. The maximum plasma concentration (*c*_max_) and time to reach the maximum plasma concentration (*t*_max_) were obtained from the observed data. The software WinNonlin (Princeton, NJ, USA), was used to calculate the area under the plasma concentration–time curve (AUC), clearance (Cl), the volume of distribution at steady state (Vss), and half-life (t1/2). The AUC was obtained by noncompartmental analysis with a linear up/log down trapezoidal method. The oral bioavailability was calculated from the oral and i.v. AUCs up to the last time point (AUC_0–6 h_) due to signals at 24 h being below the lower limit of quantification.

From the AUC, oral bioavailability (*F*) was calculated using eq 1:

$$F=\frac{{\mathrm{AUC}}_{\mathrm{p.o.}}}{{\mathrm{AUC}}_{\mathrm{i.v.}}}\times^{\;}\frac{{\mathrm{Dose}}_{\mathrm{i.v.}}}{{\mathrm{Dose}}_{\mathrm{p.o.}}}\times^{\;}100\%$$ 1

### 2.6 Two-Stage Precipitation Assay

To determine the dissolution and precipitation of both the Kolliphor solution vehicle and the SDD dispersion in citrate vehicle upon the GI passage *in vitro*, a two-stage precipitation assay was conducted which had been developed internally. Therefore, the Kolliphor vehicle containing MS4078 and the dispersion of the SDD in citrate vehicle were prepared according to the descriptions in Sections 2.5.1 and 2.5.2, respectively.

According to McConnell et al.,^23^ the volume of gastric fluid in mice is about 175 μL. Based on a dose of 10 mg/kg, with an assumed weight of ∼25 g, one animal would receive 0.25 mg compound in 0.25 mL Kolliphor vehicle, or 2.5 mg SDD powder (containing 0.25 mg MS4078) in 0.25 mL citrate vehicle. Therefore, the final concentration of MS4078 in a mouse’s stomach would be 0.25 mg/(0.175 mL+0.25 mL) = 0.588 mg/mL and, for the SDD powder, 5.88 mg/mL of solids. These concentrations were applied in the assay.

The setup of the two-stage precipitation experiments and an overview of the samples are depicted in Figure 1 and Table 2. Before starting the experiment, the vehicles (V) were stirred at 37 °C and 300 rpm for 60 min (Figure 1A). In a second step, the simulated gastric fluid pH 4 (SGF, see Section 2.2.1) was added, which resulted in the donor mixture (DM), representing the stomach of the mice (Figure 1B). The mixture was stirred at 37 °C for 15 min before starting the transfer over 20 min to the simulated intestinal compartment (Figure 1C). Three cFaSSIF concentrations were applied (1x, 5x, 10x and the dilution by the DM was accounted for by applying the double concentration (2x, 10x, 20x)). For comparison, placebo (with neat E PO instead of SDD) and blank (without E PO or SDD) experiments were conducted. The concentration of MS4078 was determined by sampling and UHPLC analysis or the turbidity of the samples was analyzed in-line using the Cary3500 (Agilent, Santa Clara, CA, USA) (Figure 1 D). The measurements were controlled by the Cary UV Workstation (Version 1.1.298, Agilent, Santa Clara, CA, USA). As a first step, the optical density (OD) of the cFaSSIF types, Blank-DM (citrate vehicle/SGF pH 4 mixture without polymer or API) and P-DM (a solution of E PO in citrate vehicle/SGF pH 4 mixture) were measured in a range of 200–800 nm (Figure S2). Since none of the constituents gave a signal at 500 nm, this wavelength was chosen for turbidity measurements. The parameters are summarized in Table S2.

**Figure 1:**
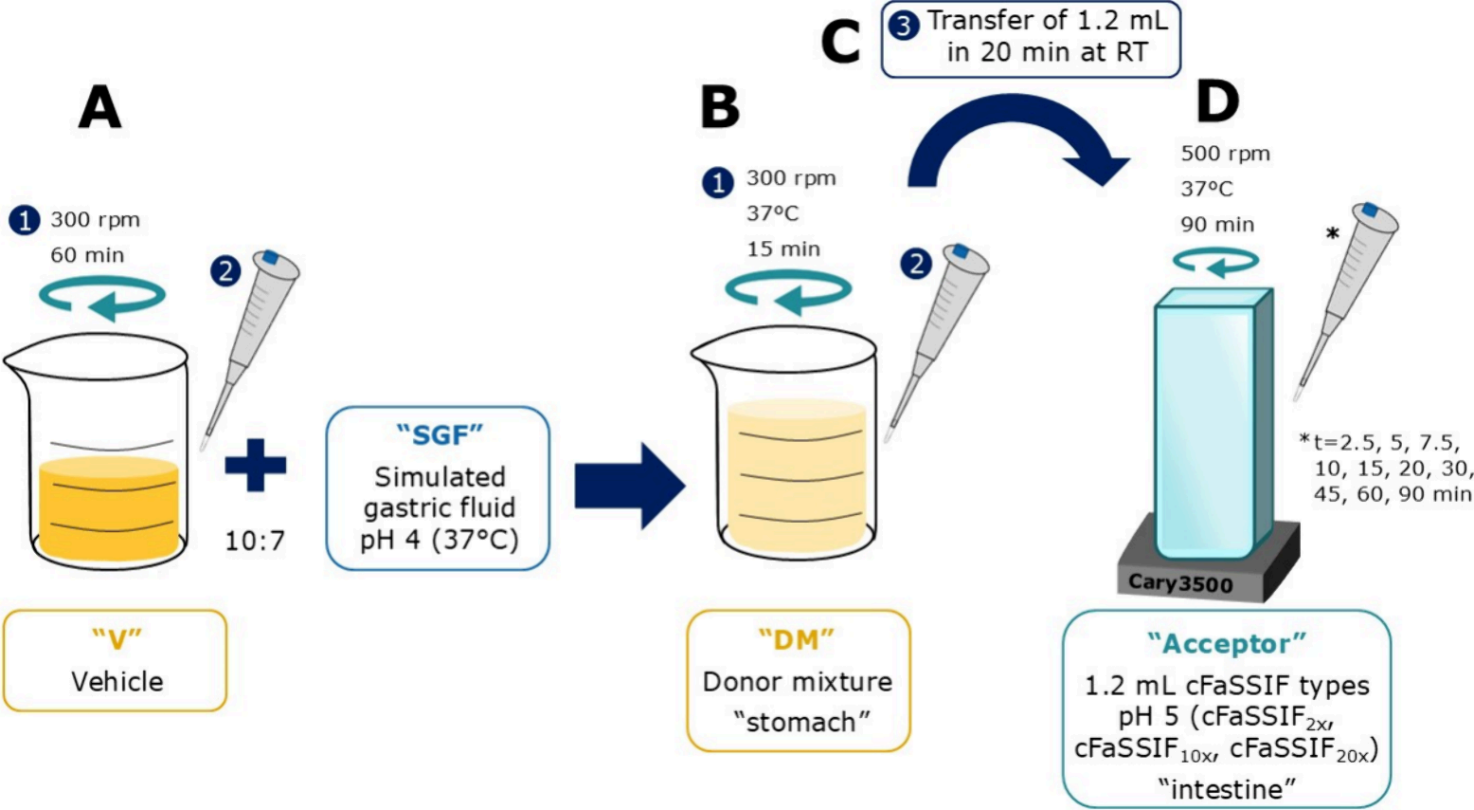
Schematic setup of the two-stage precipitation experiments. Pipette pictograms represent sample withdrawal, and the numbers indicate the timely order of the steps. All samples were centrifuged (15,000 rpm (Mikro 200 R)) for 60 s, and the supernatant was diluted with ACN and analyzed via UHPLC (see Section 2.7.1). (A) Stirring of the vehicle before the experiment. (B) Simulated gastric compartment: mixing of the vehicle and SGF. (C) Transfer of donor mixture to the simulated intestinal compartment. (D) Monitoring of dissolved MS4078 concentration or turbidity in the simulated intestinal compartment. For the transfer from the DM to the acceptor, peristaltic pumps (Reglo ICC, Ismatec, Wertheim, Germany) and transfer tubes (Tygon LMT-55, Ismatec) were used, which were calibrated before starting the experiment.

**Table 2:** Samples in the Two-Stage Precipitation Assay

Sample	Abbreviation	Formulation/material	Description
Kolliphor vehicle	K–V	API (1 mg/mL) in Kolliphor HS 15 vehicle	API in solution vehicle
Kolliphor donor mixture	K-DM	API (1 mg/mL) in Kolliphor HS 15 vehicle	K–V diluted with SGF
SDD in vehicle	SDD-V	SDD (10% MS4078, 90% E PO)	SDD dispersed in citrate vehicle (0.5% K4M, 0.25% Tween 20) (c_API_ = 1 mg/mL)
SDD donor mixture	SDD-DM	SDD (10% MS4078, 90% E PO)	SDD-V diluted with SGF
Placebo in vehicle	P–V	E PO	E PO dispersed in citrate vehicle (0.5% K4M, 0.25% Tween 20) (c_E PO_ = 9 mg/mL)
Placebo donor mixture	P-DM	E PO	P–V diluted with SGF
Blank vehicle	Blank-V	N/A	Citrate vehicle (0.5% K4M, 0.25% Tween 20)
Blank donor mixture	Blank-DM	N/A	Blank-V diluted with SGF

After the experiments, the pH values of all media, donors, and acceptors were determined. In addition, they were assessed using a light microscope (VHX-7000, Keyence, Osaka, Japan).

### 2.7 Quantitative Analysis of Samples

#### 2.7.1 UHPLC Analysis

UHPLC analysis of MS4078 is described in detail in a previous publication of our group.^7^ In brief, the samples were analyzed at 271 nm using a Waters Acquity H-Class series UHPLC system (Waters Corporation, Eschborn, Germany). A gradient was applied using ACN and water with 0.1% (V/V) trifluoracetic acid as mobile phases. Separation was realized by a Waters Acquity BEH C8 column (2.1 × 50 mm, 1.7 μm).

### 2.8 Microscopic Assessment of Turbidity

To evaluate P-DM mixtures with cFaSSIF in more detail, a microscopic assessment of samples representing the acceptor was carried out. Using the VHX-7000 microscope (Keyence, Osaka, Japan), cFaSSIF types (2x, 10x and 20x concentrated) were assessed directly after adding P-DM (c_E PO_ = 5.29 mg/mL). The resulting mixture was assessed visually, and videos were captured at magnifications of 20x-1000x of the interface between the two solutions.

### 2.9 Phase Diagram

To investigate the behavior of solutions containing different TC/PC to polymer ratios, a phase diagram was prepared with various TC/PC concentrations (0x – 20x, relative to FaSSIF-V1) in citrate-phosphate buffer pH 5 and various E PO concentrations (0–5.29 mg/mL) using the liquid-handling robot dragonfly (sptlabtech, Cambridge, UK). At first, defined TC/PC concentrations were realized by diluting stock solutions (cFaSSIF_10x_, cFaSSIF_40x_) with buffer. After homogenization (Monoshake, Variomag, Daytona Beach, FL, USA), P-DMs (c_E PO_ = 5.29 and 10.58 mg/mL) and Blank-DM were added to obtain the final E PO concentrations, followed by a second homogenization step. The optical density at 500 nm in the midpoint of the well was measured using the microplate reader Spark (Tecan, Männedorf, Switzerland) and the samples were assessed microscopically using the microscope VHX-7000.

### 2.10 Nuclear Magnetic Resonance (NMR) Studies

NMR studies were carried out to assess possible interactions of cFaSSIF and E PO. For the NMR studies, the citrate vehicle (see Section 2.4.3), SGF (see Section 2.2.1), and citrate-phosphate buffer pH 5 (see Section 2.2.2) were prepared in D_2_O instead of water. They were used for the preparation of Blank-DM, P-DM (c_E PO_ = 5.29 mg/mL), cFaSSIF_2*x*_, cFaSSIF_10x_ and cFaSSIF_20x_ (see Sections 2.6 and 2.2.2). All ^1^H and 2D diffusion-ordered spectroscopy (DOSY) NMR studies were performed on a Bruker 500 MHz Avance III spectrometer (Bruker, Billerica, MA, USA) equipped with a helium cryocooled H&F BBO probe at 298 K sample temperature. All saturation transfer difference (STD) NMR studies were performed on a Bruker 700 MHz Avance III spectrometer (Bruker) equipped with a helium cryocooled TCI probe at 303 K sample temperature.

#### 2.10.1 1D ^1^H NMR Spectroscopy

The 1D ^1^H NMR spectra were recorded by 30° excitation over a spectral width of 25 ppm. The FID was digitized with 64k data points, zero-filled to 128k data points, and multiplied by an exponential function (lb 0.3) before Fourier transformation. A total of 32 spectra were accumulated with a relaxation delay of 1 s.

#### 2.10.2 Saturation Transfer Difference (STD) NMR

Saturation transfer difference (STD) spectra were recorded to check for weak intermolecular interaction of phosphatidylcholine and E PO. Therefore, selective saturation of the phosphatidylcholine signal at 5.26 ppm was performed by using a *SINC1.100* pulse with an excitation bandwidth of 100 Hz. A total of 128 spectra were accumulated, and the relaxation delay was set to 2 s. The nonsaturated spectrum was subtracted from the saturated spectrum.

#### 2.10.3 2D Diffusion-Ordered Spectroscopy (DOSY)

Diffusion-ordered spectroscopy (DOSY) was used to investigate the self-diffusion of all components in the solution. Therefore, cFaSSIF types and again combinations of P-DM and Blank-DM and the different cFaSSIF types were measured. DOSY spectra were recorded with the Bruker standard pulse sequence *dstebpgp3s* using bipolar gradient pulses of a total length of 2.4 ms (little delta), a diffusion delay of 100 ms (big delta), and a 32-step linear gradient ramp (5% to 95% gradient strength). A total of 16 spectra were accumulated for each gradient step. The 2D DOSY plot was calculated using the Bruker software Dynamics Centre (V 2.8.4) by application of an exponential decay fitting routine.

## 3 Results

### 3.1 Solubility of MS4078

The solubility of MS4078 in buffer and different cFaSSIF types after 24 h is shown in Figure 2. Very low concentrations were measured in the buffer (∼2 μg/mL) alone, whereas the concentration of dissolved API was dependent on the TC/PC concentration in the cFaSSIF types. The higher the TC/PC concentration, the more MS4078 dissolved.

**Figure 2:**
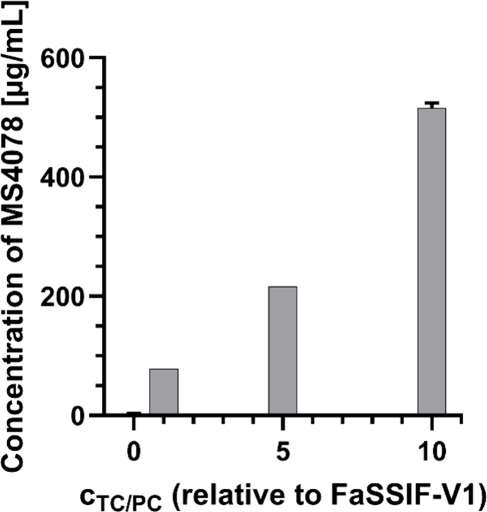
Solubility of MS4078 in citrate-phosphate buffer and different cFaSSIF types after 24 h (Mean ± SD, *n* = 3).

### 3.2 Pharmacokinetic Study in Mice

After promising dissolution results of the SDD formulation containing 90% E PO and 10% MS4078 in our previous work,^7^ a pharmacokinetic study using this formulation in mice was carried out in the present study. The aim was to evaluate if the oral bioavailability of the compound would be improved by the enabling formulation.

For the PK study, a vehicle for the homogeneous dispersion of the SDD formulation was necessary and a 100 mM citrate vehicle pH 3 containing 0.5% Methocel and 0.25% Tween 20 was chosen which yielded a constant concentration of MS4078 between 15 and 60 min after its initial dissolution (Figure S3). Furthermore, a reference PK study was done with a solution vehicle where MS4078 was soluble at 1 mg/mL. The vehicle screening identified 5% DMSO/20% Kolliphor HS15 in water as the vehicle of choice which was administered orally and intravenously to mice.

The results of the pharmacokinetic studies of MS4078 in Kolliphor vehicle (K–V; 10 mg/kg po and 0.5 mg/kg (iv) and as an SDD formulation dispersed in citrate vehicle (SDD-V; 10 mg/kg po) are shown in Figure 3 and Table 3. All pharmacokinetic parameters of the SDD formulation were calculated based on the results from two animals since the third animal needed to be excluded from the study according to the attending veterinarian. The root cause for the clinical symptoms could not finally be clarified and an impact of the vehicle cannot be excluded.

**Figure 3:**
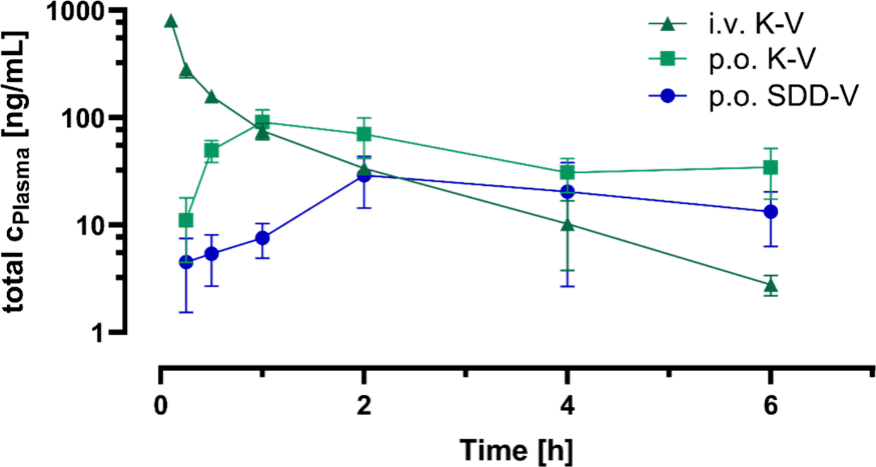
Plasma concentration–time profiles of MS4078 in mice after intravenous and oral administration (Mean ± SD, *n* = 2 (SDD-V) and *n* = 3 (K–V)). Total concentrations are displayed. 0.5 mg/kg MS4078 in the Kolliphor vehicle (K–V) were administered intravenously; 10 mg/kg MS4078 were administered orally, either as K–V or after dispersion of the SDD in a citrate vehicle containing 0.5% Methocel and 0.25% Tween 20 (SDD-V). The concentration of MS4078 was below the limit of quantification at the 24 h sampling point for all tested formulations.

**Table 3:** Pharmacokinetic Parameters after a Single Administration of K–V (i.v. and p.o.) and SDD-V (p.o.) to Mice (Mean ± SD, n = 3; n = 2 in SDD-V Arm (*))

Sample	Dose [mg/kg]	*c*_max_ [ng/mL]	*t*_max_ [h]	AUC _last_ [h*ng/mL]	*t*_1/2_ [h]	F [%]
K–V (i.v.)	0.5	808.3 ± 98.7	0.1	405 ± 39	1.11	N/A
K–V (p.o.)	10	91.4 ± 27.1	1	284 ± 65	3.9	3.5
SDD-V (p.o.)*	10	29.1 ± 14.6	2	106 ± 65	3.6	1.3

The intravenous study revealed a moderate *in vivo* total clearance (1.22 L/h/kg; 20% mouse liver blood flow) and volume of distribution (Vss = 1.05 L/kg). For assessing oral bioavailability, dose-normalized mean AUC_last(0–6h)_ values of the oral arms of the study were related to the mean of intravenous data. For MS4078 in K–V an oral bioavailability of 3.5% was calculated, whereas a bioavailability of 1.3% was obtained for MS4078 in SDD-V. However, such small differences may only be interpreted in considerably larger groups of animals. Consequently, a similarly low exposure was found after administration of the two formulations. *T*_max_ was slightly later for the SDD formulation than for K–V (2 h versus 1 h).

### 3.3 Two-Stage Precipitation Assay: Verum

To simulate the dissolution and precipitation behavior of the two formulations (K–V, SDD-V) during GI passage, a two-stage precipitation assay was carried out. Therefore, the pH of SGF and cFaSSIF was adjusted to mimic physiological conditions in mice,^23^ and different TC/PC concentrations were applied to cover a broad range (TC: 3–30 mM, PC: 0.75- 7.5 mM). The results of the assays in different cFaSSIF types are represented in Figure 4 and the pH of the solutions and compartments are summarized in Table S3. In the graph, *c*_tot_ is the total concentration of MS4078 that has been transferred (dotted line), and c_s,DM_ is the extrapolated concentration (dashed line) calculated based on the measured dissolved part of MS4078 in the DM (simulating the stomach) before the transfer.

**Figure 4:**
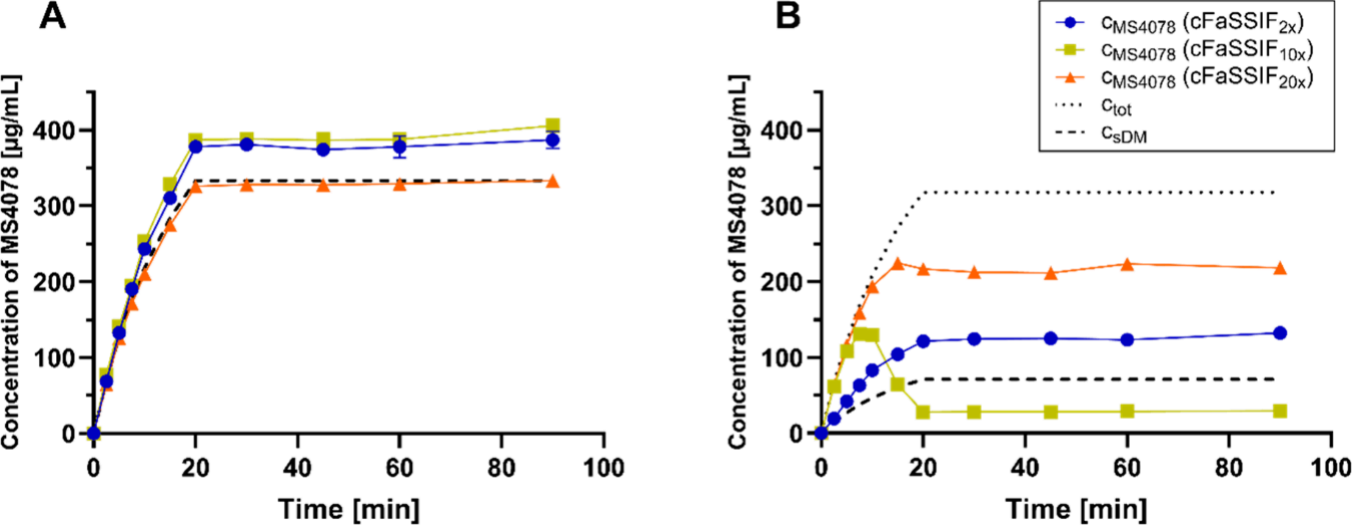
Dissolved MS4078 in the acceptor compartment after transfer of (A) K-DM (Mean ± SD, *n* = 3). (B) SDD-DM to different cFaSSIF types (Mean ± SD, *n* = 3). *c*_tot_: maximum feasible MS4078 concentration, c_s,DM_: extrapolated MS4078 concentration, based on the dissolved part in the DM.

The entire amount of MS4078 was dissolved in K-DM and throughout the transfer to the acceptor (Figure 4A). In this graph, the extrapolated concentration (c_s,DM_) equals the maximum feasible concentration (*c*_tot_) as the entire amount of API was in solution in K-DM. In comparison to the extrapolated profile, slightly higher values were found in the samples. The reason for the deviation from c_sDM_ might be based on volume contraction during dilution for UHPLC analysis or evaporation from the UHPLC samples. The concentrations were double-checked with an inline derivative spectroscopic method (based on Lehmann et al.^24^), where the results matched perfectly with the extrapolated profile (data not shown). From both analytical techniques, it can be concluded that the entire amount of API was in solution during the two-stage precipitation assay, independent of the TC/PC concentration. This unexpected result may be attributed to Kolliphor HS15, which is a solubilizing agent and was applied in relatively high concentrations above its critical micelle concentration (CMC) even in the acceptor.^25^ Despite various pH shifts and dilution steps, the vehicle was very potent and stabilized MS4078 throughout the entire assay.

In contrast, MS4078 was only partially dissolved in the SDD-DM, therefore the extrapolated profile c_s,DM_ is lower than the maximum feasible concentration *c*_tot_. Different profiles were detected during the transfer experiments to the different cFaSSIF media (Figure 4B). For cFaSSIF_2*x*_, within the first 2.5 min, the concentration of dissolved API matched the extrapolated curve. Afterward, the concentration started to exceed the extrapolated value which indicates a dissolution of MS4078 from particles in the acceptor cuvette. After 20 min, the concentration plateaued at 130 μg/mL.

Interestingly, for cFaSSIF_10x_, a completely different profile was observed. Within the first 5 min the concentration followed *c*_tot_ (the maximum). Since MS4078 was only partially dissolved in the DM, upon transfer to the acceptor medium, the remaining API must have dissolved immediately. Starting at 5–7.5 min, a deviation from the maximum was observed, and a peak concentration was reached after 10 min, followed by a decrease until the 20 min sampling point. Here, only 30 μg/mL could be maintained in solution until the end of the experiment.

In cFaSSIF_20x_ (the highest TC/PC concentration), again a different profile was obtained. This time, the measured concentration started to deviate from *c*_tot_ after 10–15 min. However, no pronounced precipitation was observed (as it had been for cFaSSIF_10x_), but a plateau was reached.

Since all parameters were kept identical, except for the concentration of TC/PC in the different cFaSSIF media, effects of the solubilizing excipients Methocel and Tween 20, which were constituents of the dispersion vehicle of the SDD, could be excluded. They may have affected the dissolved MS4078 concentration in the donor mixture and during the transfer of the DM, as their concentration increased in the acceptor cuvette along with the dispersed SDD, however, the effect would be identical in all experiments. Consequently, the different supersaturation profiles of MS4078 were attributed to the different TC/PC concentrations in the assays.

### 3.4 Two-Stage Precipitation Assay: Placebo

During the diverse verum two-stage precipitation assays described above, the solutions in the cuvettes appeared turbid at different time points and, in some instances, became clear again throughout the assay. These observations may be caused by precipitation or droplet formation. Interestingly, the turbidity was not in accordance with the decrease in API concentration. It was hypothesized, that either the polymer E PO, or one of the other constituents of the solution precipitated (and redissolved) or emulsified during the assay. Hence, placebo two-stage assays were conducted with P-DM and Blank-DM to evaluate the polymer separately from the compound and the optical density (OD) was measured inline (Figure 5).

**Figure 5:**
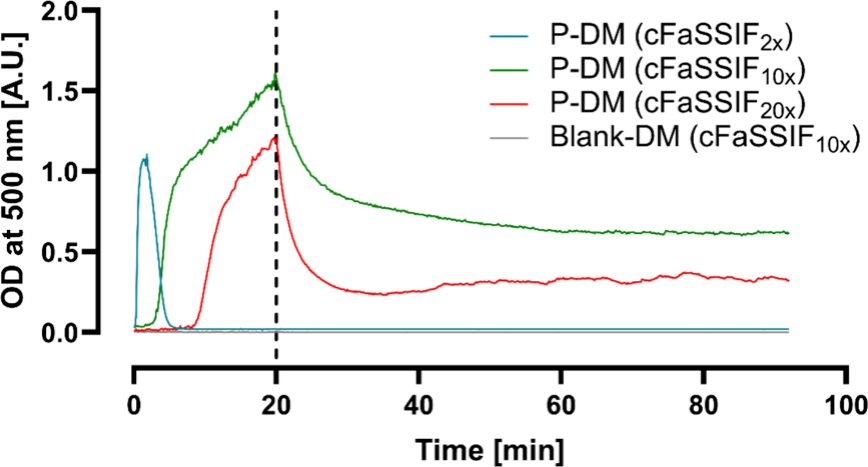
Turbidity measurements at 500 nm of the two-stage precipitation assays of P-DM to different cFaSSIF types (Mean, *n* = 3) and Blank-DM to cFaSSIF_10x_ (*n* = 1, representative for the transfer to the different cFaSSIF types). The end of the transfer from the donor solution is indicated by the dashed line at *t* = 20 min.

The blank samples were clear (OD < 0.02 A.U.) throughout the experiment. In contrast, an increase in OD, i.e. an increase in turbidity, was observed for placebo solutions in all three cFaSSIF types. Consequently, the turbidity was caused by E PO, probably by precipitation of the polymer due to pH shifts (pH 3.3 (DM) to pH 5.0 (acceptor cuvette) (Table S3)) or phase separation. For cFaSSIF_2*x*_, the increase in absorbance occurred immediately, followed by a steep decrease within the first 6 min. Afterward, a low signal remained, indicating that the solution had become clear. For cFaSSIF_10x_ and cFaSSIF_20x_ the increase in OD was observed after ∼2.5 min and ∼8.5 min, respectively. With the end of the DM transfer (t = 20 min), the OD decreased abruptly, but a turbidity signal remained until the end. The solutions in the acceptor cuvettes were assessed microscopically after the completion of the assay. Clear solutions without signs of precipitate or droplets were found for P-DM + cFaSSIF_2*x*_, whereas droplets were detected in the cuvettes containing either cFaSSIF_10x_ or cFaSSIF_20x_ (Figure S4).

To evaluate if precipitation occurred initially in combination with phase separation, the addition of a droplet of P-DM to cFaSSIF directly on a microscope slide was monitored in detail under the microscope. All tested samples turned macroscopically turbid upon the addition of E PO solution (Figure S5). The interface of the two solutions was assessed, where only droplets formed (Figure S5). Consequently, the turbidity was solely caused by phase separation.

Based on these findings in the placebo samples, the acceptor compartment after transfer of the SDD to cFaSSIF_10x_ and cFaSSIF_20x_ was checked for droplets. Here, yellow droplets were visible (Figure 6). The color was probably caused by the yellow compound, MS4078. These observations hint at a (at least partial) dissolution of the API in the droplets.

**Figure 6:**
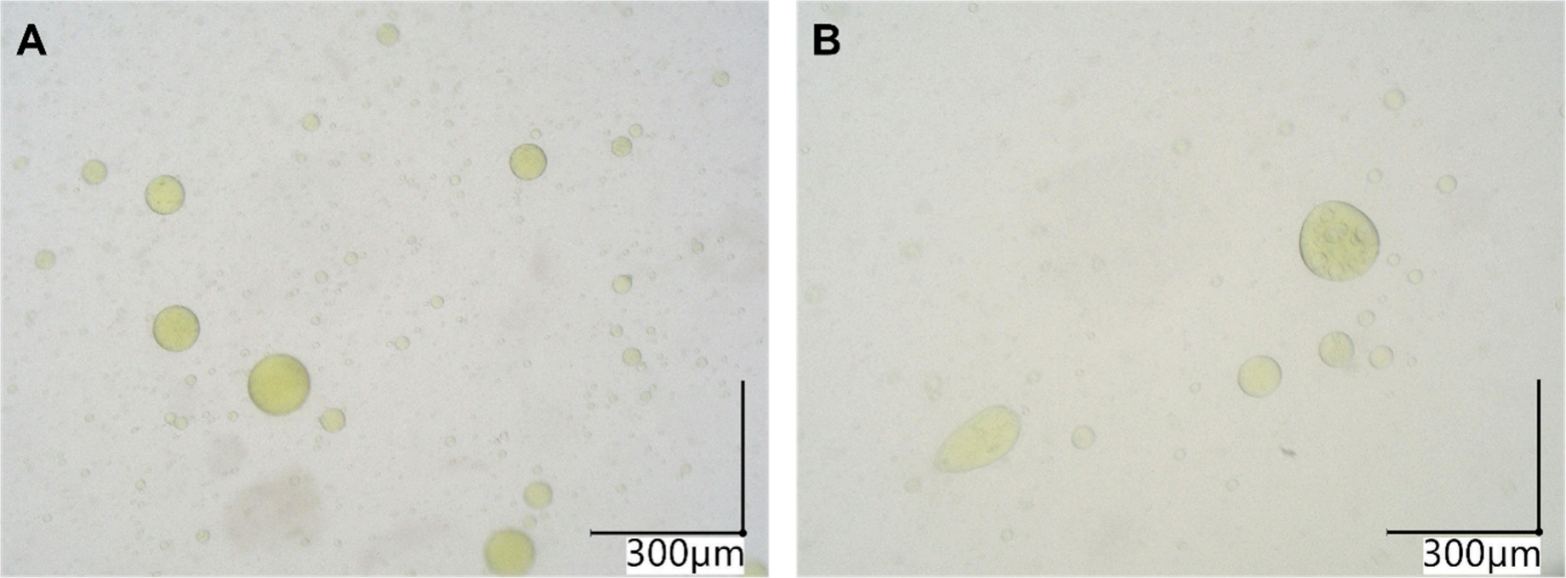
Representative microscopic pictures of acceptor solutions after transfer of SDD-DM in (A) cFaSSIF_10x_ and (B) cFaSSIF_20x_ at 200× magnification.

### 3.5 Phase Diagram

To identify the limit for a permanent droplet formation, a phase diagram using various concentrations of TC/PC and E PO was prepared. The concentration of Methocel and Tween 20 was kept constant to minimize their influence and vary only the ratio of TC/PC and E PO. Before mixing, all samples that contained TC/PC and polymer, appeared turbid (data not shown). After homogenization, the decisive parameter was the presence of droplets, i.e. a stable phase separation. Droplets formed by mixing cFaSSIF_2.5x_ and P-DM containing 1.32 mg/mL E PO, whereas for higher concentrated E PO solutions (10.58 mg/mL), permanent phase separation was only obtained for 8x or higher concentrated cFaSSIF (Figure 7). Hence, it was concluded from the results that the ratio of TC/PC to E PO has an impact on the phase separation.

**Figure 7:**
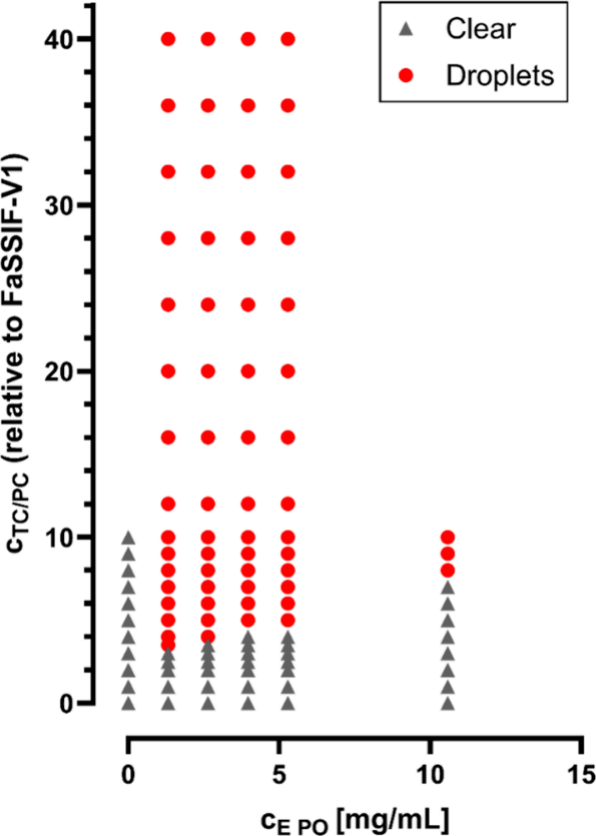
Phase diagram of P-DM and cFaSSIF mixtures. The concentrations of both TC/PC in cFasSIF and E PO were not adjusted for the dilution factor (1:1).

### 3.6 NMR Studies

^1^H NMR investigations were performed for a better structural understanding of the droplets and the supernatant. Therefore, samples representing the acceptor cuvettes of blank and placebo assays were used. In the first step, the specificity of ^1^H NMR regarding the supernatant in the turbid samples was checked. As already shown, the turbidity was caused by small, microscopically visible droplets. These droplets are typically too large aggregates to be observed using solution state ^1^H NMR spectroscopy. The ^1^H NMR spectra of the turbid solutions in cFaSSIF_10x_ and cFaSSIF_20x_ and the clear supernatant were identical in terms of line positions and line widths (Figure S6). Hence, the method can be considered specific concerning the supernatant.

In a second step, the ^1^H NMR spectra of Blank-DM, P-DM, cFaSSIF_10x_ and P-DM + different cFaSSIF types were measured (Figure S7). The ^1^H NMR spectra of P-DM and Blank-DM + cFaSSIF_10x_ showed a high degree of signal overlap. Nevertheless, there were two spectral regions in which a clear distinction between E PO and cFaSSIF could be made. The signal at 5.25 ppm could be assigned to the double bond of PC and the signal at 2.94 ppm to the dimethyl amine group of E PO (Figure S1). Additional cFaSSIF signals were mostly located around 0.6–2.3 ppm (e.g., TC methyl group at 0.64 ppm,^26^ Figure S1). The ^1^H NMR spectra of the placebo samples showed a very indicative behavior. The line widths of the cFaSSIF components were strongly broadened in the clear P-DM + cFaSSIF_2*x*_ sample. This can be caused by various factors (e.g., slower diffusion, increase in viscosity). The line width of the citrate signals did not change; hence, the viscosity of the system was not altered significantly. In the context of the system investigated here, the line broadening was most likely caused by a pronounced increase in the hydrodynamic volume, e.g. by intermolecular interaction between E PO and cFaSSIF or by the formation of an aggregate. Furthermore, in the spectra of the turbid samples (P-DM + cFaSSIF_10x_ and cFaSSIF_20x_), the E PO signal at 2.94 ppm disappeared and the cFaSSIF signal intensity decreased. The simultaneous formation of the turbidity and the signal decrease of E PO and PC strongly indicate a phase transition in which E PO, TC, and PC were removed from the solution.

The obtained ^1^H NMR spectra could also be used to get a better understanding of the droplet composition. The signals of citrate (2.7 and 2.8 ppm) served as an internal reference to evaluate the relative composition of the components, as the citrate concentration was identical for all samples (Figure 8A). When comparing Blank-DM + cFaSSIF_10x_ with P-DM + cFaSSIF_10x_, the area of the phosphatidylcholine signal was reduced by ∼75% and of taurocholate by ∼43%. Hence, it can be concluded that the droplets were mainly composed of E PO and PC, while TC was present in lower concentrations. When comparing P-DM + cFaSSIF_20x_ with P-DM + cFaSSIF_10x_, one will expect an increase in concentration by 100% for PC and TC if the turbid phase was saturated with the cFaSSIF components. This holds true for TC, but for PC the increase was only ∼90% (Figure 8A). Consequently, PC was still partly incorporated in the turbid phase.

**Figure 8:**
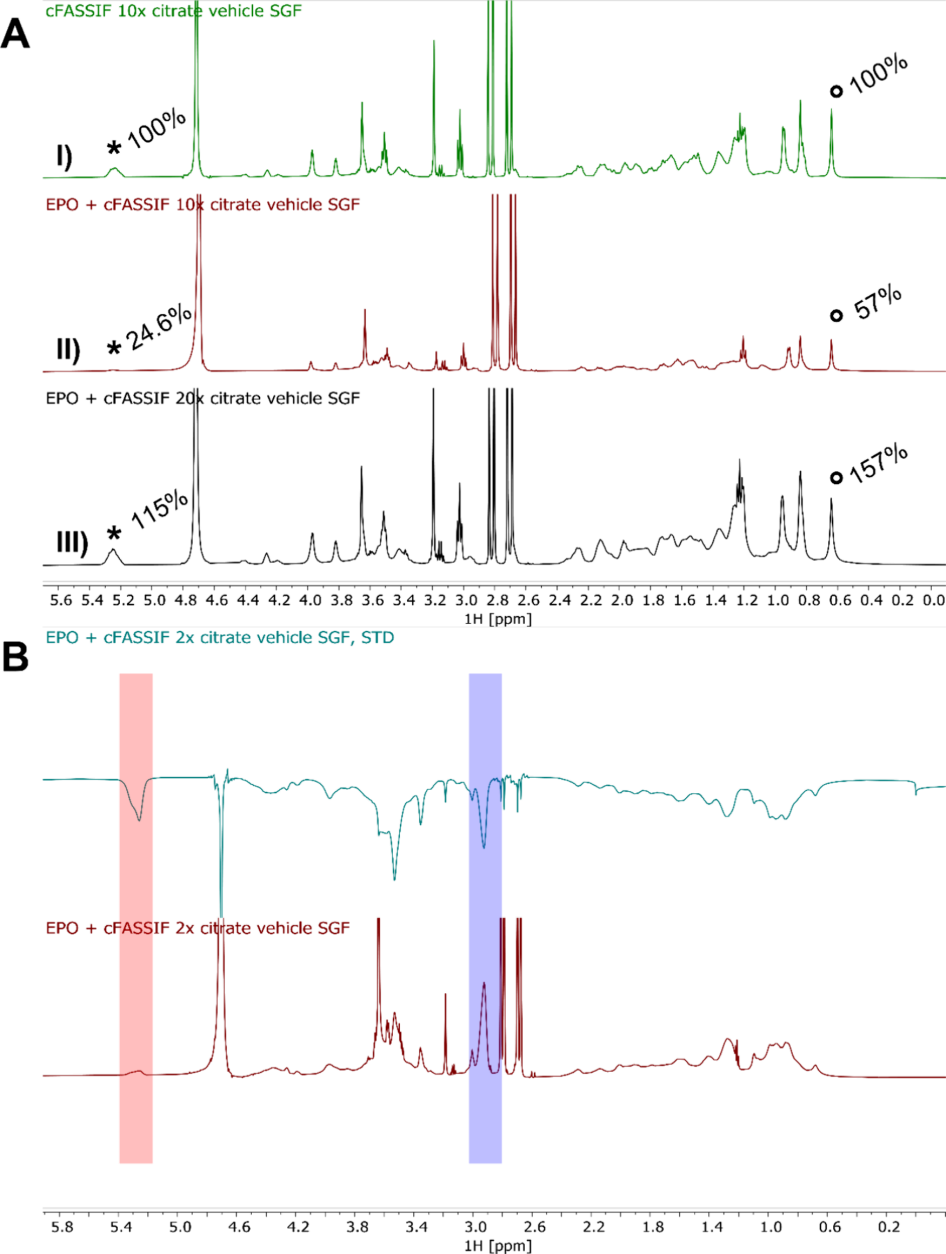
(A) Normalized ^1^H NMR spectra of (I) Blank-DM in cFaSSIF_10x_ and (II) P-DM in cFaSSIF_10x_ and III) in cFaSSIF_20x_. The positions and relative areas of the relevant signals of PC (*) and TC (°) are indicated. (B) Difference spectrum (top) and control spectrum (bottom) from the STD NMR study of P-DM in cFaSSIF_2*x*_. The PC signal (red area) was saturated, and the saturation transfer to the E PO signal (blue area) was evaluated.

As observed, at the phase transition point mainly E PO and PC were extracted from solution into the droplets. This may originate from a weak intermolecular interaction between these two components. Saturation transfer difference (STD) spectra were recorded for the clear P-DM in cFaSSIF_2*x*_ to substantiate this assumption. This technique is typically used to detect weak intermolecular interactions in solution.^27–29^ For this purpose, the PC signal at 5.25 ppm was selectively saturated. This saturation was indeed transferred to E PO as observed by a strong response signal at 2.94 ppm (Figure 8B), thereby proving the weak interaction between these two components. It is important to mention that TC and the polymer may also interact, but a detailed investigation was not possible due to the signal position in the spectrum.

In an additional experiment, the self-diffusion of all components in the solution was investigated by DOSY (diffusion-ordered spectroscopy). The self-diffusion constant depends on the hydrodynamic volume of a compound.^30^ An increase in the hydrodynamic volume, which would be indicated by a decrease in the diffusion constant, is assumed as the main origin of the strong line broadening of the cFaSSIF component in the P-DM + cFaSSIF_2*x*_ sample. This behavior was clearly seen in the DOSY spectra of the clear P-DM, Blank-DM + cFaSSIF_2*x*_ and P-DM + cFaSSIF_2*x*_ (Figure 9) samples. E PO showed the lowest diffusion constant (∼10^–9.8^ to 10^–10^ m^2^/s), which remained nearly unchanged in the presence of cFaSSIF. This is also in accordance with the observed very high line width in the ^1^H NMR. In contrast, cFaSSIF showed a more complex diffusion behavior. Mainly two different phases in the range 10^–9.4^ and 10^–9.7^ m^2^/s were observed in the blank sample which is in agreement with literature.^31,32^ But in the presence of E PO, the DOSY spectrum clearly showed a transition to a single phase with a lower diffusion constant of ∼10^–9.8^ m^2^/s. This shift in the diffusion proves the increase in the hydrodynamic volume of the cFaSSIF components and explains the pronounced increase in ^1^H NMR line widths of the P-DM + cFaSSIF_2*x*_ sample. The turbid P-DM + cFaSSIF_10x_ and P-DM + cFaSSIF_20x_ samples showed a comparable, complex DOSY profile like the Blank-DM + cFaSSIF sample (Figure S8). The absolute values of the diffusion constant were little shifted, which may be caused by the difference in the stoichiometry of the cFaSSIF components.

**Figure 9:**
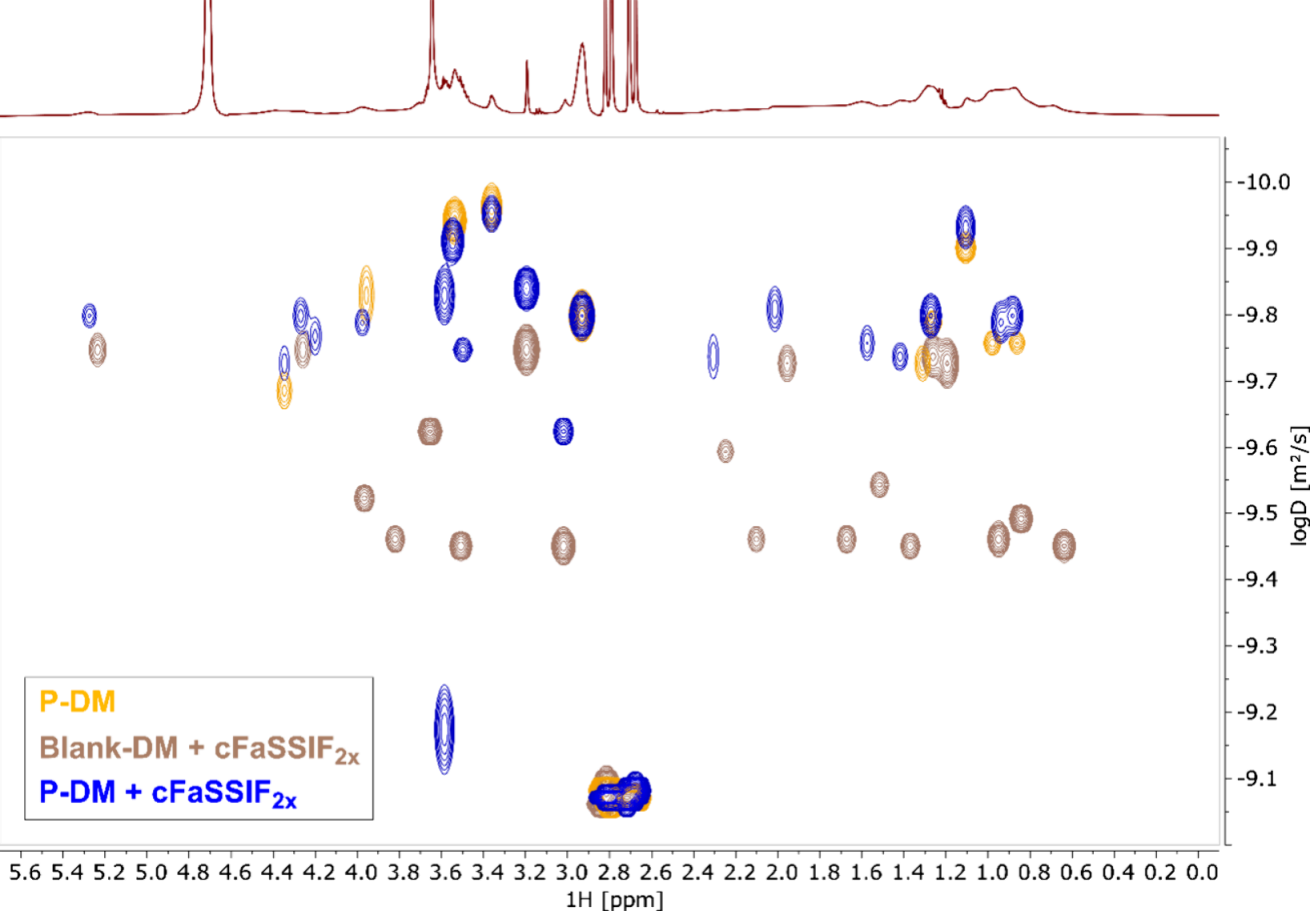
2D DOSY spectrum of P-DM, Blank-DM in cFaSSIF_2*x*_, and P-DM in cFaSSIF_2*x*_.

## 4 Discussion

The rapid advancement of Vepdegestrant (ARV-471) and other clinical PROTAC candidates prove the paramount potential of PROTACs and the need for new therapeutics in their respective fields.^1,33^ In most cases, the poor solubility of these PROTACs is addressed by employing different formulation strategies like cyclodextrins or amorphization with or without polymeric carriers.^5,34^ Amorphous solid dispersions have shown promise in enhancing the solubility of various PROTAC molecules *in vitro*.^7,36^ However, further investigation is needed to evaluate the effectiveness of this concept *in vivo.*

In our previous study, the amorphous spray-dried formulation of MS4078 in E PO as a polymeric carrier, provided a >70fold supersaturation compared to the neat compound *in vitro*.^7^ Therefore, it was chosen for a pharmacokinetic study in mice. Due to the size of this preclinical species, an administration as powder in capsule is not possible, hence, the SDD powder was given as oral gavage after dispersion in a citrate vehicle containing 0.5% Methocel and 0.25% Tween 20 vehicle (SDD-V). As benchmark, a solution formulation (1 mg/mL MS4078, 5% DMSO and 20% Kolliphor HS15 in water, K–V) was additionally administered intravenously to determine relevant PK parameters and to judge oral bioavailability. It was observed, that *c*_max_ was reached later for the SDD formulation compared to K–V (Table 3). This observation was in good accordance with the *in vitro* dissolution profile, where the API was released over 1.5 h.^7^ For K–V, a bioavailability of only 3.5% was found. Unexpectedly, a similar low bioavailability for the SDD was observed.

The disappointing bioavailability of the SDD had not been anticipated after the promising results of the dissolution test in FaSSIF. A deeper investigation of the supersaturation and precipitation of MS4078 from both formulations was carried out *in vitro* with a special focus on the pH and bile salt condition. Therefore, a two-stage precipitation assay, which resembles the human gastrointestinal passage, was adapted to represent the intestine of mice. According to McConnell et al.,^23^ pH values are ∼4 in the stomach and ∼5 in the intestine of mice. In compliance with this study, SGF pH 4 and a citrate-phosphate buffer pH 5 were prepared. The buffer was chosen due to the broad buffer range, a suitable buffer capacity at pH 5, citrate already being present in the assay, and previous implementation for the simulation of intestinal fluid of mice.^37^

Bile salts (FaSSIF-V1: taurocholate (TC), human reference: 3 mM) and phospholipids (FaSSIF-V1: phosphatidylcholine (PC), human reference: 0.75 mM) are employed in dissolution media to resemble the mixed micelles in the intestine, that are essential for the solubilization and uptake of hydrophobic and lipidic substances.^15,38^ Unfortunately, the concentrations and composition of intestinal fluids in mice are not well investigated. To cover a broad range, three different TC/PC concentrations were included by adding different amounts of 3F powder to prepare cFaSSIF (Table 1). They comprised a human equivalent (low bile salt concentration, 3 mM (1x)) and an elevated concentration (30 mM (10x)), which would be approximately present in rats, a species with high bile salt abundance in the intestine.^19,39^ As a reference point, a third concentration (15 mM (5x)) was prepared, which had been used in previous mouse-adjusted *in vitro* assays.^37^ The 1:1 dilution during the precipitation assay was accounted for by the preparation of media with a double concentration (2x, 10x, 20x).

In the adjusted two-stage precipitation assay, both K–V and SDD-V were tested to evaluate and compare their behavior under mice-relevant conditions. Despite the lack of a strong precipitation inhibitor, its poor solubility in low TC/PC media (S­(FaSSIF-V1)=20 μg/mL, S­(cFaSSIF_1*x*_)=78 μg/mL), the pH changes, and dilution steps during the assay, MS4078 could entirely be maintained in solution throughout the simulated GI passage of K–V (Figure 4A). This may be attributed to the solubilizing agent Kolliphor HS15, and, probably, the compound was also entirely dissolved in the intestine of the mice. Nevertheless, only 3.5% of MS4078 were bioavailable.

Unlike K–V, the supersaturation provided by SDD-V was dependent on the TC/PC concentration (Figure 4B). However, no clear correlation between these factors was identified. Besides, turbidity of the samples was noticed during the experiments which did not correlate with the decrease in the API concentration. During time-resolved turbidity measurements of placebo samples in the same assay, two interesting findings were made: First, the turbidity was caused by the polymer (see Section 3.4 and Figure 5). Second, a dependency on the TC/PC concentration in the acceptor medium was observed. In detail, the turbidity was temporary in cFaSSIF_2*x*_, whereas it persisted in cFaSSIF_10x_ and cFaSSIF_20x_ until the end of the assay (Figure 5). A thorough microscopic assessment revealed that not precipitation but phase separation with droplet formation was the cause (Figure S5). Thus, a phase diagram was set up. The lower the concentration of E PO, the less TC/PC was necessary to permanently form droplets and vice versa (Figure 7). Consequently, it was concluded that the ratio of TC/PC to E PO is essential.

The results from the phase diagram were compared to the data obtained from the placebo two-stage precipitation assays (a relevant cutout is shown in Figure 10). As the P-DM was pumped over 20 min, the ratio of E PO to TC/PC in the acceptor changed constantly. Due to the increased TC/PC concentration in cFaSSIF_10x_ and cFaSSIF_20x_, the phase separation was observed later (i.e., at higher E PO concentrations) compared to cFaSSIF_2*x*_. In cFaSSIF_2*x*_, the ratio for phase separation was reached almost immediately, but with increasing E PO concentration, the solution became clear again. These findings are in perfect accordance with the data from the phase diagram – above a certain ratio no permanent droplet formation was observed (Figure 10, indicated by the arrows). Assessment of the solutions in the cuvettes after the placebo assays confirmed the observations – in P-DM + cFaSSIF_10x_ and P-DM + cFaSSIF_20x_ droplets were found (Figure S4) whereas P-DM + cFaSSIF_2*x*_ was clear.

**Figure 10:**
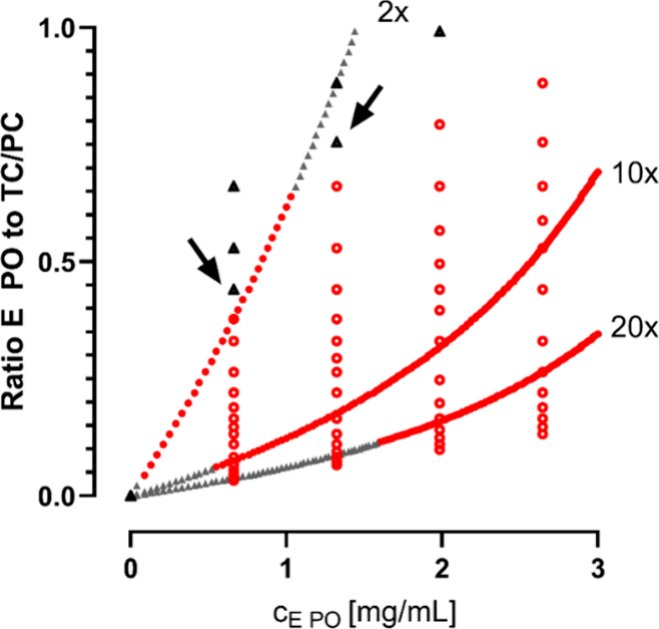
Comparison of turbidity data from phase diagram (Figure 7) and two-stage precipitation assays (Figure 5). The black and gray triangles represent clear solutions, whereas the red circles indicate droplet formation in the respective solution. Open circles/black triangles are the results from the phase diagram experiment, whereas filled circles/gray triangles represent data points obtained from the two-stage assays to the indicated cFaSSIF type.

The composition of droplets was characterized by ^1^H NMR studies. The aggregates consisted of TC, PC, and E PO (Figures 8A and 9) and an interaction of PC and E PO could be proven (Figure 8B). These findings are supported by the studies by Schlauersbach et al, who investigated the influence of several polymers on bile salt interacting drugs and found a structural change in FaSSIF micelles in the presence of E PO due to interactions.^13^ Hence, they hypothesized that, in dependency on the E PO concentration, the drugs would either be pushed out of the micelles, or at high concentrations, they would be taken up into the aggregates and the free drug concentration would be reduced. However, a detailed investigation of the aggregates or the impact on drug solubilization was not conducted. Additional information about the type of interaction, the relevant constituents, characterization of the aggregates and resulting droplets, and a phase diagram with phase separation limits were provided in the present study (Figures 7, 8, and 9).

The impact on the drug concentration may be evaluated utilizing the SDD-DM results from the two-stage precipitation assays. Microscopy showed that the droplets were also present in the verum cuvettes at high TC/PC concentrations (Figure 6). Hence, MS4078 did not interfere with the interplay of E PO and TC/PC but was in contrast accumulated in the droplet phase (see Section 3.4).

In cFaSSIF_2*x*_, the time point of compound dissolution in the acceptor compartment (see Section 3.3) coincided with the disappearance of the turbidity, i.e. the redissolution of the aggregates that formed the droplets (Figure 11). As E PO and TC/PC were again available in the aqueous phase, they solubilized the compound and prevented precipitation. Compared to the solubility data of the neat API in the medium (Figure 2), a supersaturation was provided by the enabling formulation.

**Figure 11:**
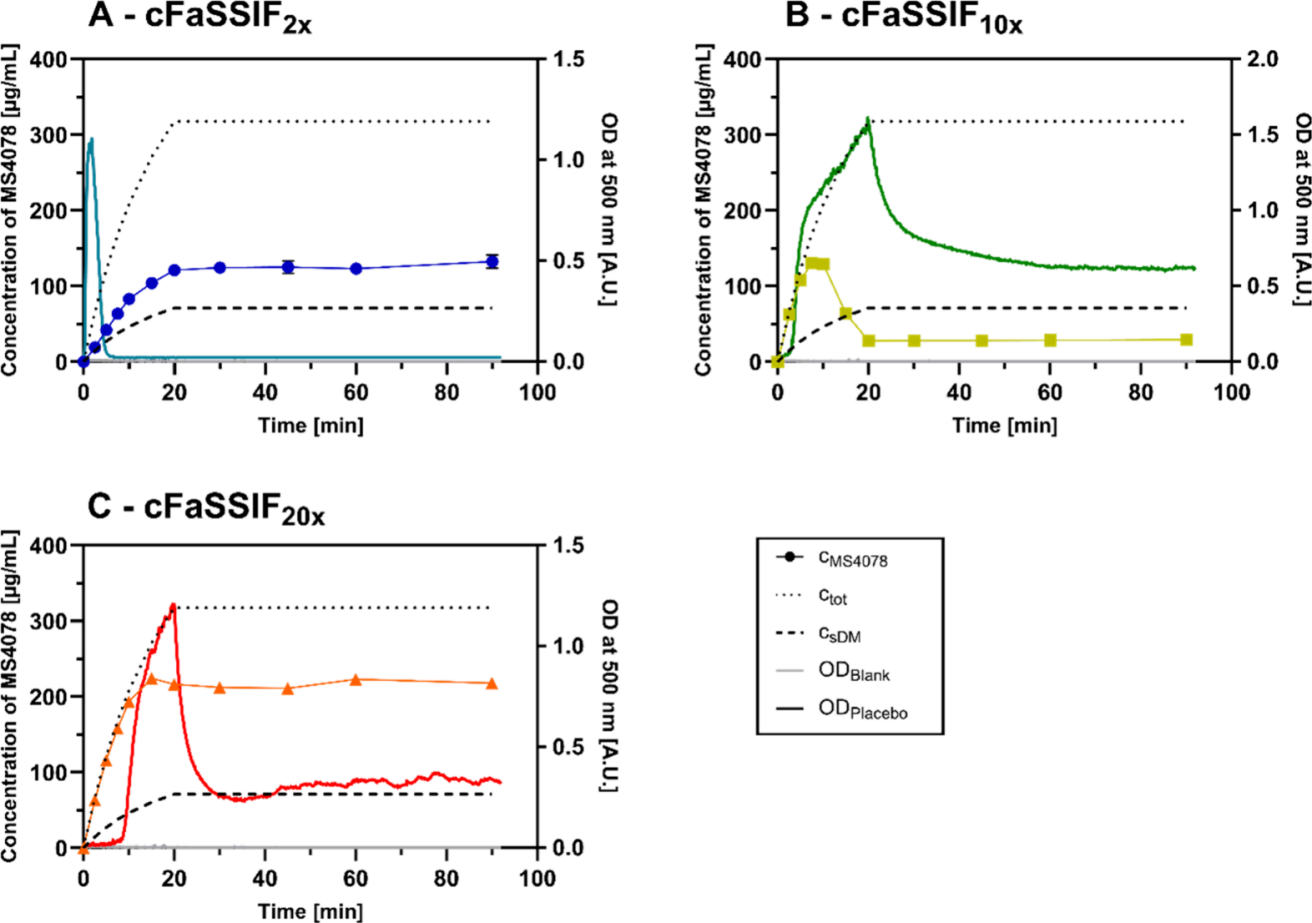
Overlay of the turbidity data of placebo two-stage precipitation assays at 500 nm (OD_Placebo_, Figure 5) and the concentration of MS4078 (c_MS4078_; Mean ± SD, *n* = 3, Figure 4) after transfer of SDD-DM in the acceptor compartment containing (A) cFaSSIF_2*x*_, (B) cFaSSIF_10x_, and (C) cFaSSIF_20x_.

In general, TC/PC act as solubilizers, therefore it is reasonable to anticipate that more compound dissolves in media with an increased amount of these molecules. This was confirmed by the solubility of MS4078 in cFaSSIF types (Figure 2) and is additionally visible in the transfer to cFaSSIF_20x_ (Figure 11). At first, MS4078 was entirely dissolved but with the onset of phase separation, a deviation was observed, and a plateau was maintained. With the transition of TC/PC and the polymer into the droplet phase, MS4078 followed along. The concentration of MS4078 in the transfer experiment after the phase separation matches perfectly with the solubility in 5x concentrated cFaSSIF (∼220 μg/mL (Figure 2)). However, in this case, 10x TC/PC would be theoretically present after a 1:1 dilution of cFaSSIF_20x_ with P-DM. This discrepancy can be explained by the NMR studies: TC/PC transitioned into the droplet phase and only a TC/PC concentration resembling approximately 5x FaSSIF remained in the aqueous phase of P-DM + cFaSSIF_20x_ (Figure 8A). Thus, it can be concluded, that the phase separation impacts the dissolution and that the stabilization of MS4078 occurred mainly by solubilization in the remaining colloidal cFaSSIF structures and not the polymer, as most of the latter had transitioned into the droplet phase.

Taking a closer look at the compound concentration during the transfer to cFaSSIF_10x_, this influence is clearly visible. Due to the high amount of TC/PC in the aqueous phase at the beginning, the entire API was in solution until the droplet phase formed (Figure 11). Consequently, the API concentration in the aqueous phase dropped drastically with the phase separation as neither enough polymer nor cFaSSIF micelles were present to stabilize a supersaturation.

Not only TC/PC, and E PO are solubilizers in the acceptor compartment, but Methocel and Tween 20 were transferred along with the API. Both may influence the phase diagram and the solubility and stabilization of MS4078. Their concentration and the transfer rate were kept constant, thus their effect on the dissolved amount of MS4078 was comparable in all setups. Taking into account the last paragraphs, and the lack of phase separation in Blank-DM (Figures 5, 7, and 10), a minor effect of Methocel and Tween was concluded, whereas the discussed interplay of E PO, TC and PC was predominant.

In summary, in dependency on the TC/PC concentration, not only the formulation was changed from a dispersion to an emulsion, but also the stabilization of MS4078 in solution was affected, due to the availability of the solubilizers and precipitation inhibitors – taurocholate, phosphatidylcholine and E PO – in the aqueous phase. Transferring these findings to *in vivo*, it is very probable that the bioavailability would also be affected. This hypothesis is supported by the results of Schlauersbach et al, who connected increased compound entrapment into colloids of E PO and bile salts to reduced passage over a semipermeable membrane^13^ and to changes in the *in vivo* performance^40^ These findings raise a controversial point in the view of E PO as a solubilizing agent and polymeric carrier for ASDs and a critical point in the selection of species for PK studies. Furthermore, they show that it is imperative to interpret results from PK studies concerning the species which must also be considered during extrapolation.

In the present case, unfortunately, due to incomplete information about physiological conditions in mice, a final definition of the cause for the poor *in vivo* performance is not possible. Nevertheless, it can be concluded, that the entire amount of API was stabilized in solution by K–V, but still, a similar low bioavailability as for the SDD was found. This shows that solubility enhancement alone may not be sufficient to address the bioavailability challenges of MS4078, but further work is needed to identify the hurdle for improved bioavailability.

## 5 Conclusions

During the development of enabling formulation systems for poorly soluble compounds like e.g. the model PROTAC MS4078, the *in vivo* performance is essential. A promising enabling formulation, the spray-dried formulation containing 90% E PO as a polymeric carrier and 10% MS4078 was tested in a PK study in mice. Unfortunately, the promising dissolution results did not translate to *in vivo*. A root cause analysis via a two-stage precipitation assay revealed a performance dependency of the E PO SDD on the taurocholate/phosphatidylcholine concentration. The supersaturation and stabilization of MS4078 were influenced by droplet formation and interaction of TC/PC and E PO.

Besides, a thorough characterization of the interaction of TC/PC (and therefore FaSSIF) with E PO was conducted. Based on these results, the following points should be considered for E PO as a polymeric carrier. First, E PO and TC/PC interact and form an emulsion system depending on their ratio. The limits were identified in this study. Second, these interactions might influence the bioavailability and need to be considered especially in animal species with high bile salt and phospholipid concentrations. Third, representative results from *in vitro* assays are only obtained if biorelevant media containing bile salts and phospholipids are applied. In addition, the outcome of this study emphasizes the importance of detailed information on *in vivo* conditions and on excipients *in vitro* and *in vivo* for a well-grounded decision on the additives in the formulation.

## Supplementary Material



## Notes

Additional experimental results; (Figures S1) chemical structures (MS4078, E PO, PC, and TC); (Figure S2) absorbance spectra (200–800 nm) of cFaSSIF types, P-DM, and Blank-DM; (Figure S3) free MS4078 concentration after the dispersion of SDD in citrate vehicle; (Figure S4) microscopic pictures of acceptor cuvettes; (Figure S5) microscopic pictures of cFaSSIF types and E PO solution; (Figure S6) ^1^H NMR spectra (P-DM + cFaSSIF_10x_/cFaSSIF_20x_) of turbid sample vs clear supernatant; (Figure S7) ^1^H NMR spectra (P-DM (+cFaSSIF types), Blank-DM + cFaSSIF_10x_); (Figure S8) DOSY spectra (P-DM (+cFaSSIF types), Blank-DM (+cFaSSIF_2x_)); (Table S1) composition of citrate-phosphate buffer pH 5; (Table S2) parameters of in-line analytics using the Cary3500; and (Table S3) pH of media in two-stage precipitation assays, donor, and acceptor compartments (DOCX)

## Abbreviations

1D: two-dimensional
2D: one-dimensional
ACN: acetonitrile
API: active pharmaceutical ingredient
AUC: area under the curve
ASD: amorphous solid dispersion
Blank-V/Blank-DM: citrate vehicle/citrate vehicle + SGF
*c*_max_: maximum concentration
CMC: critical micelle concentration
c_sDM_: extrapolated concentration based on the dissolved part of MS4078 in the donor mixture
c_TC/PC_: concentration of bile salts and phosphatidylcholine relative to FaSSIF-V1 (100%)
*c*_tot_: maximum feasible concentration of MS4078 in two-stage precipitation assays
D_2_O: deuterium oxide
DCM: dichloromethane
DM: donor mixture in two-stage precipitation assays (ten parts of vehicle plus seven parts of simulated gastric fluid)
DMSO: dimethyl sulfoxide
DOSY: diffusion-ordered NMR spectroscopy
E PO: Eudragit E PO, cationic methacrylate-copolymer
(c)­FaSSIF: (citrate-based) fasted state simulated intestinal fluid, the index indicates the concentration of 3F powder for preparation (relative to FaSSIF-V1)
GI: gastro-intestinal
IVIC: *in vivo*-*in vitro*-correlation
K–V/K-DM: API solution in Kolliphor vehicle/donor mixture
MeOH: methanol
NaCl: sodium chloride
NaOH: sodium hydroxide
NMR: nuclear magnetic resonance
OD: optical density
PC: phosphatidylcholine, a phospholipid of lecithin
PK: pharmacokinetic
PoI: protein of interest, target of a PROTAC
PROTACs: proteolysis targeting chimeras, bifunctional degraders
P-V/P-DM: placebo (E PO) dispersed in citrate vehicle/donor mixture
SDD: spray-dried dispersion
SDD-V/SDD-DM: SDD dispersed in citrate vehicle/donor mixture
SGF: simulated gastric fluid
STD: saturation transfer difference NMR spectroscopy
*t*_max_: time point of cmax
*V*_ss_: volume of distribution at steady state
