# Supplementary material for: PROTAC Enabling Formulation In Vivo: Implications of the Polymeric Carrier Eudragit E PO
Source: Mol Pharm. 2025 Aug 24;22(10):5845–59. doi: 10.1021/acs.molpharmaceut.5c00303 (PMC12505254; doi:10.1021/acs.molpharmaceut.5c00303)
Supplement: Supplementary file 1 [file mp5c00303_si_001.docx]

PROTAC^®^ enabling formulation *in vivo* – implications of the polymeric carrier Eudragit^®^ E PO

Nicole Hofmann^a,b^, Florian Johann^a^, Katharina Krollik^a^, Andreas Marx^c^, Heide Marika Duevel^d^, Marc Lecomte^d^, Meike Harms^a^, Karsten Mäder^b^

^a^ Global Drug Product Development, Orals Development, the Healthcare Business of Merck KGaA, Frankfurter Straße 250, 64293 Darmstadt, Germany

^b^ Institute of Pharmacy, Faculty I of Natural Sciences, Martin Luther University Halle-Wittenberg, Kurt-Mothes-Strasse 3, 06120 Halle (Saale), Germany

^c^ Site Management Lab Services, Merck KGaA, Frankfurter Straße 250, 64293 Darmstadt, Germany

^d^ Global Research and Development, NCE DMPK, the Healthcare Business of Merck KGaA, Frankfurter Straße 250, 64293 Darmstadt, Germany

**Supplementary material**

Figures


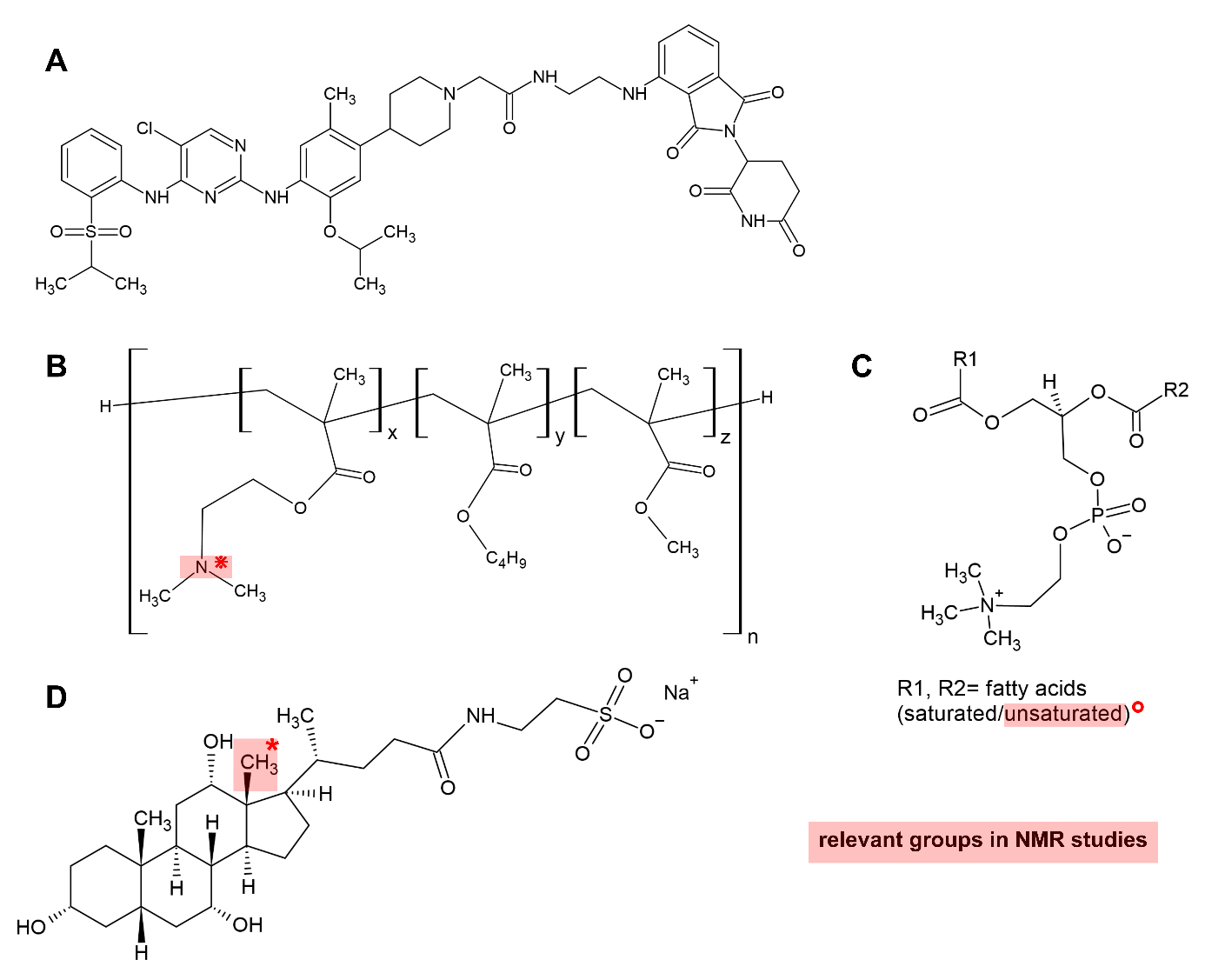


**Figure S1.** Chemical structures of A) MS4078, B) E PO, C) lecithin (phosphatidylcholine), D) sodium taurocholate.


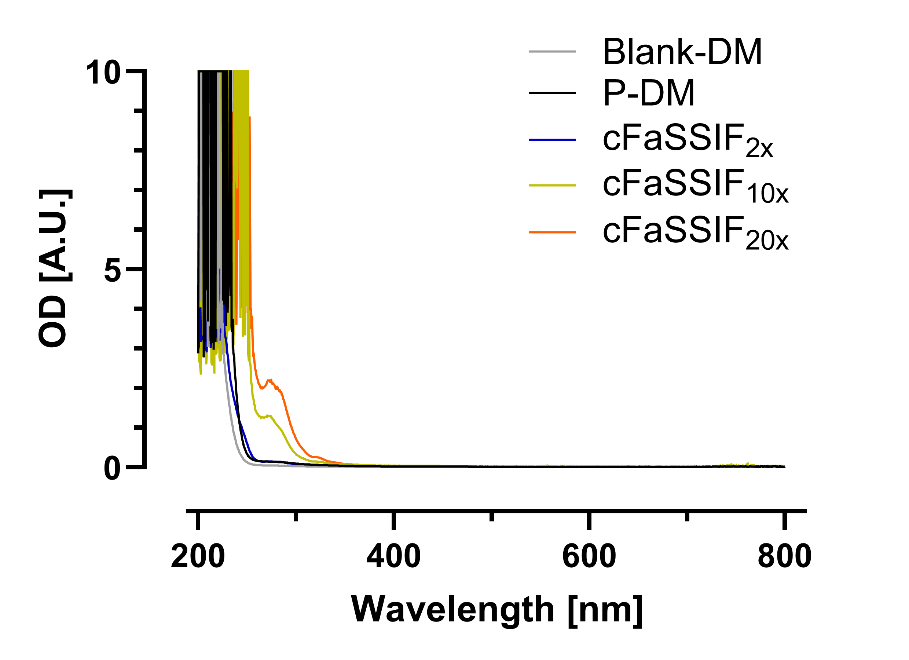


**Figure S2.** Spectra of cFaSSIF types, Blank-DM, P-DM in a range of 200-800 nm.

**Figure S3.** Free concentration of MS4078 after the dispersion of SDD formulation in citrate vehicle (100 mM citrate pH 3 + 0.5% Methocel + 0.25% Tween 20). (n=1).


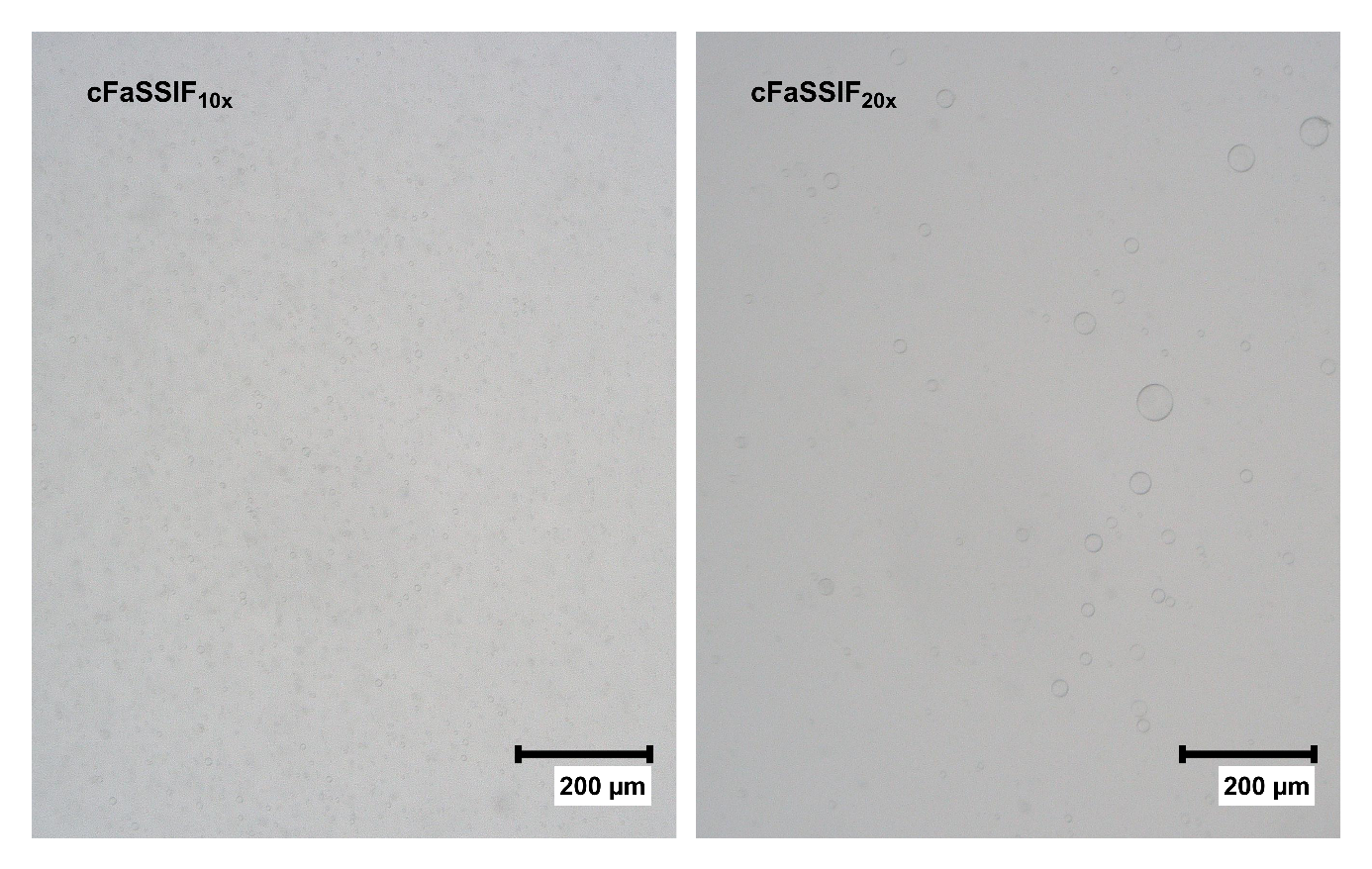


**Figure S4.** Microscopic pictures of solutions in acceptor cuvettes after transfer of P-DM at 200x magnification.


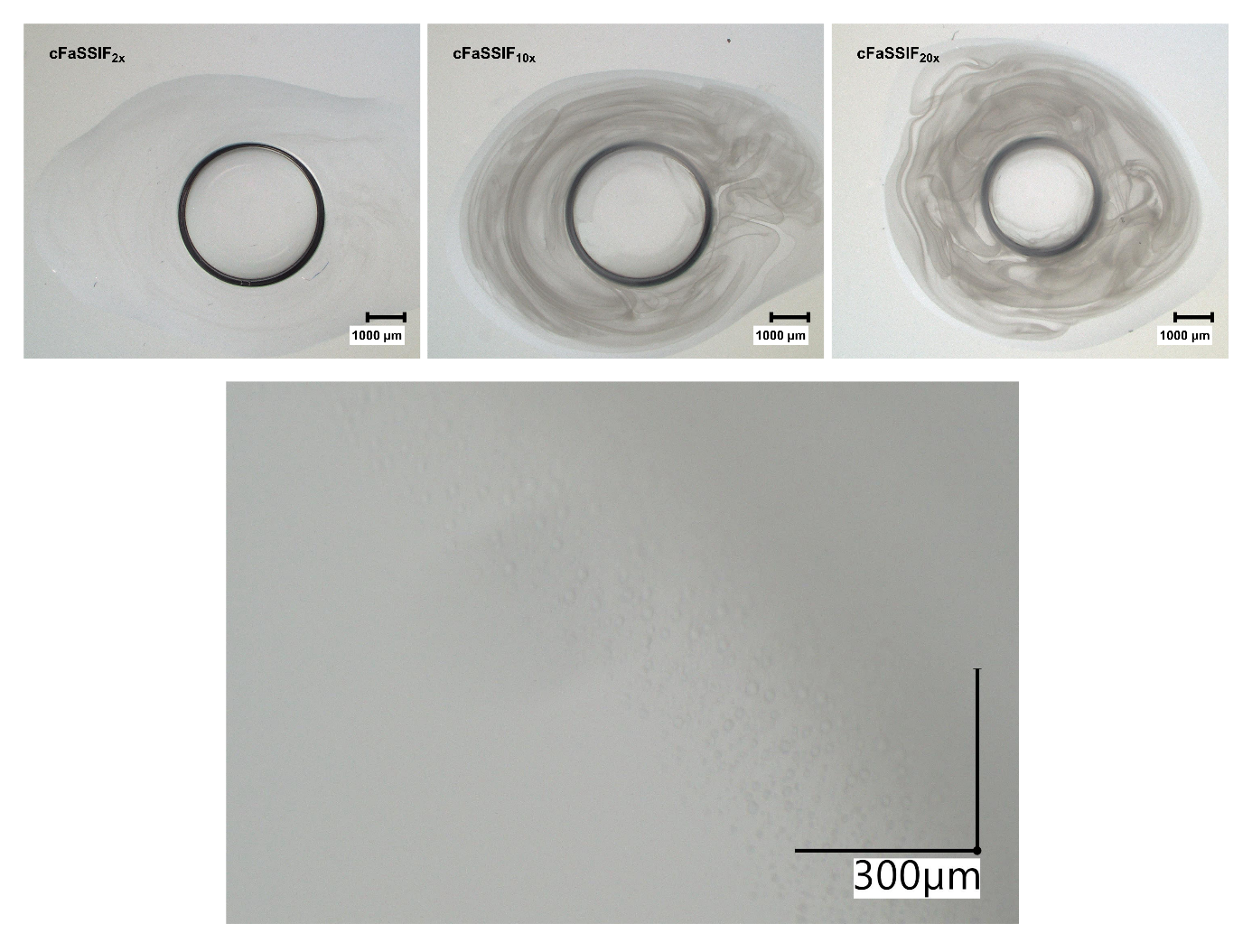


**Figure S5.** Top: Microscopic pictures of cFaSSIF types after addition of a droplet of E PO solution at 20x magnification. Bottom: Microscopic picture of interface of solutions after addition of E PO solution to cFaSSIF_10x_ at 500x magnification.


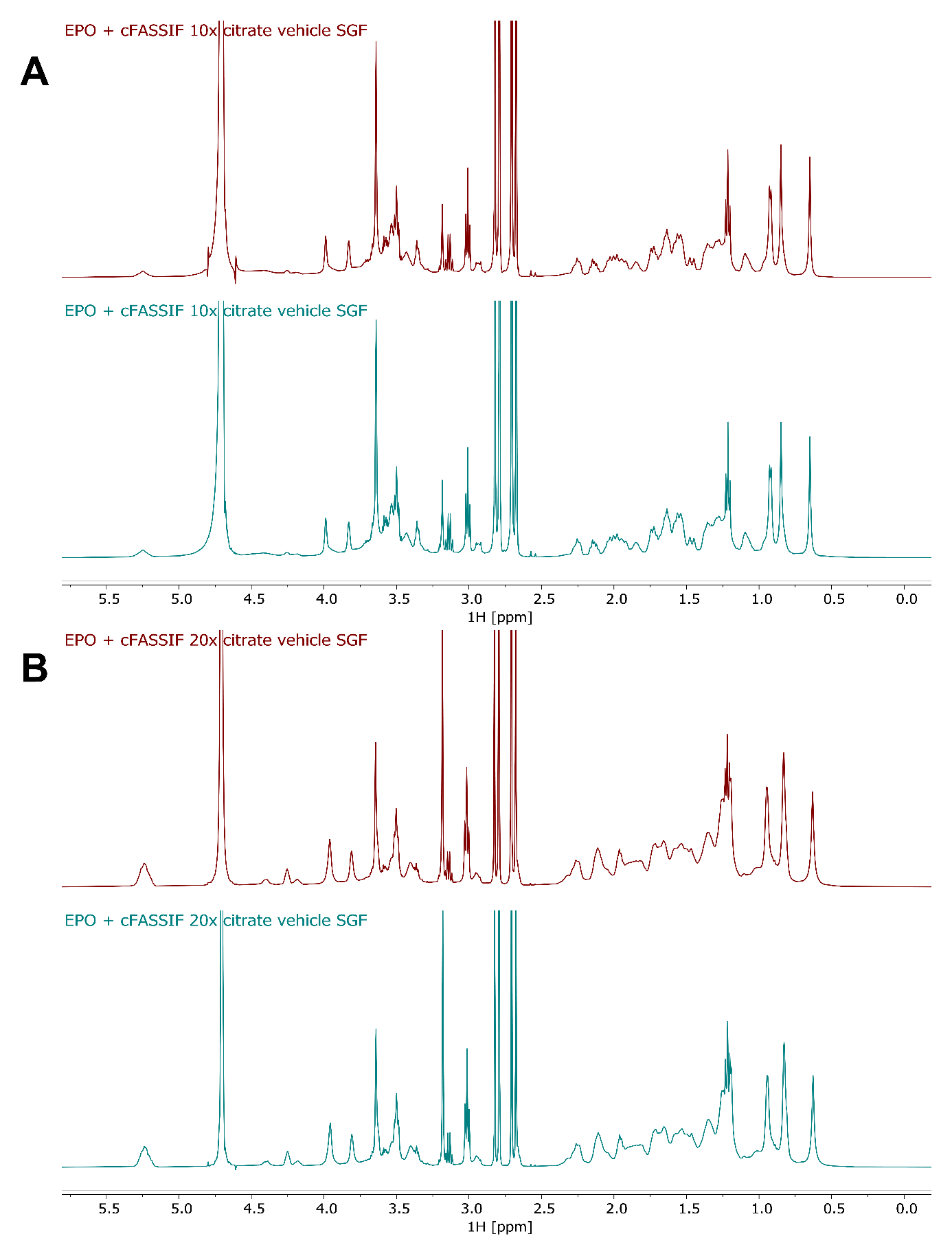


**Figure S6.** ^1^H NMR spectra of E PO in citrate vehicle/SGF mixture (P-DM) with A) cFaSSIF_10x_ and B) cFaSSIF_20x_ of turbid sample with emulsion droplets (top) and clear supernatant after separation of the droplet phase (bottom).


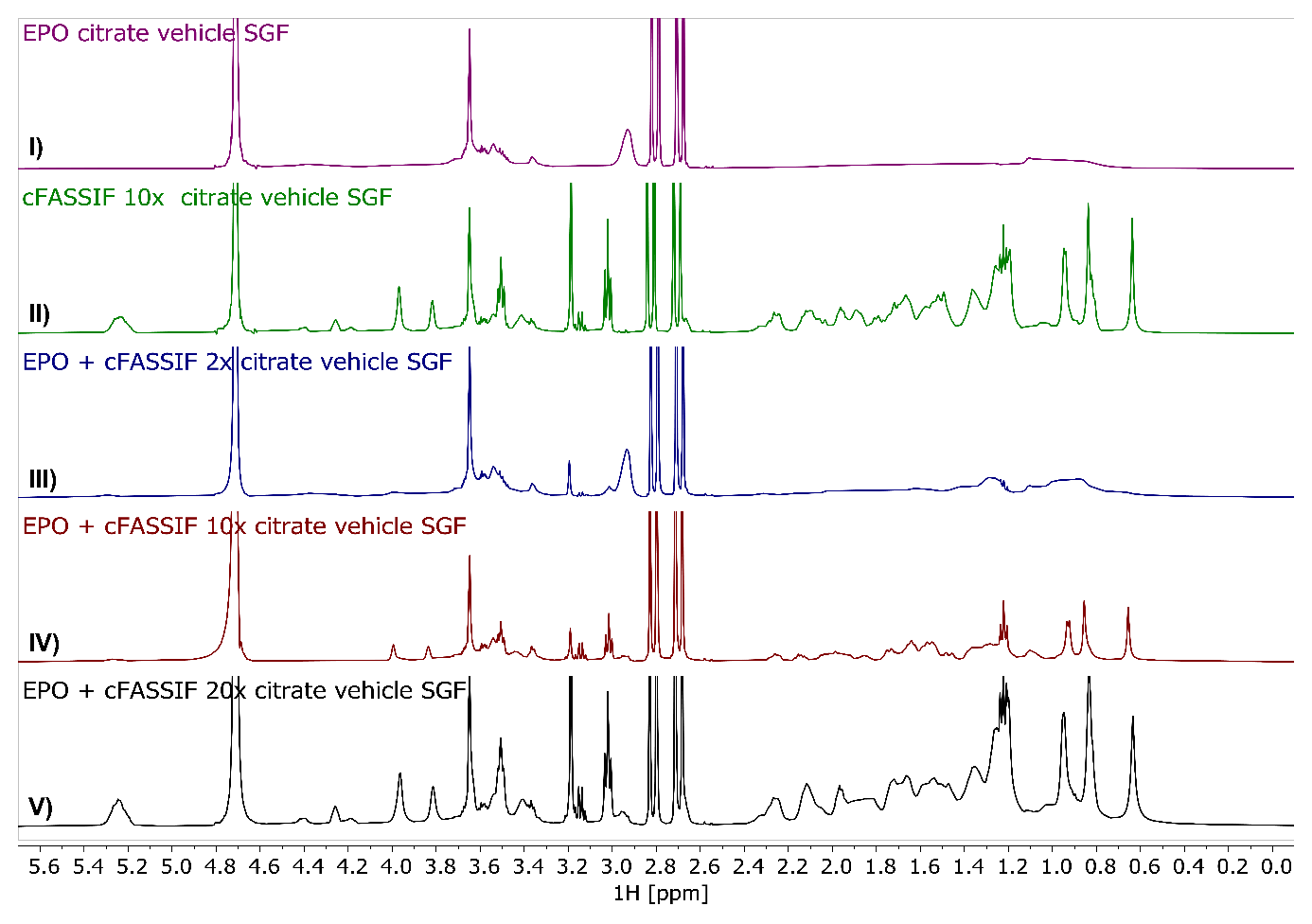


**Figure S7.** ^1^H NMR spectra of I) P-DM, II) Blank-DM in cFaSSIF_10x_ and III)-V) the combination of P-DM and cFaSSIF types.


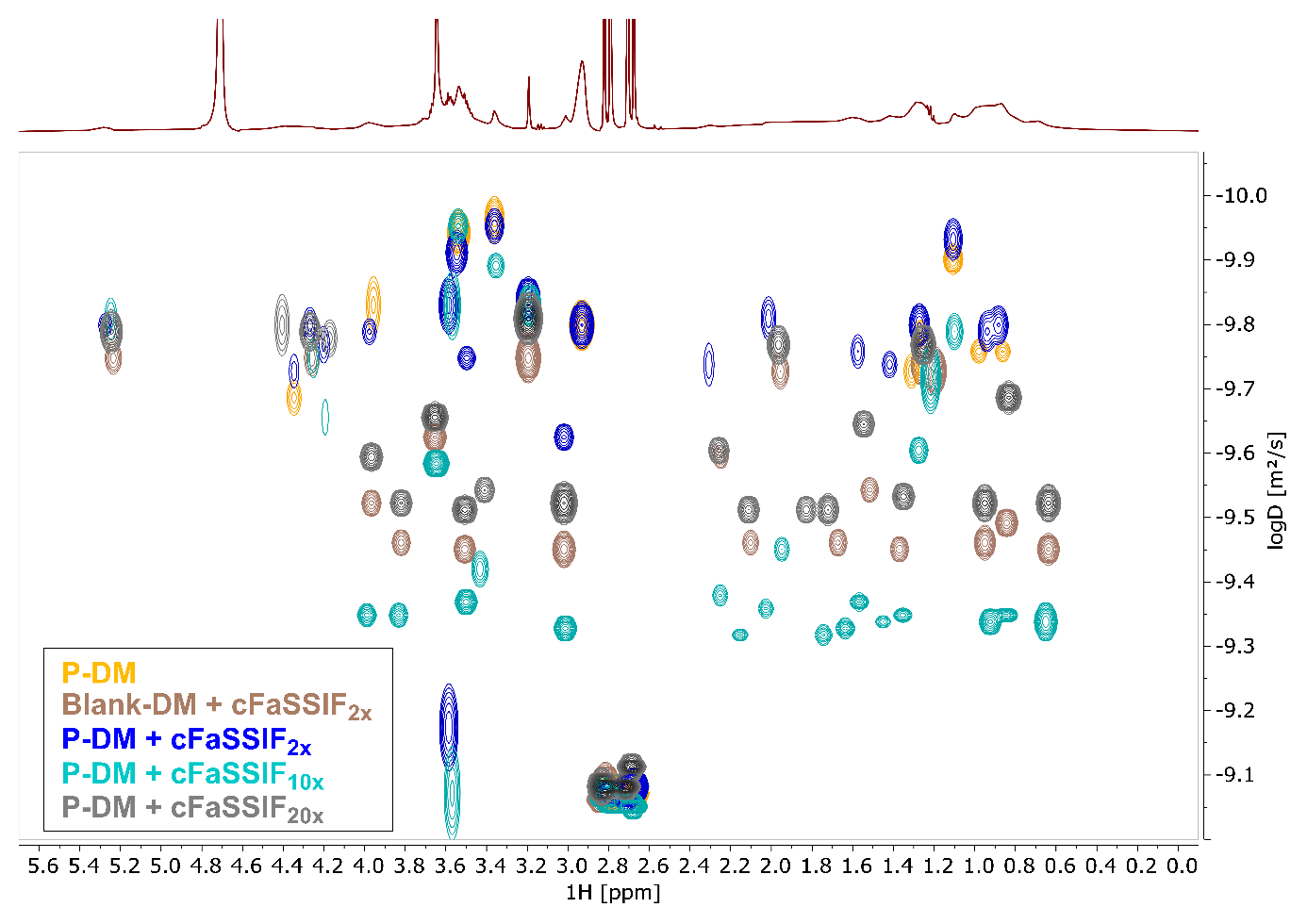


**Figure S8.** 2D DOSY spectra of P-DM and Blank-DM + cFaSSIF_2x_ as references, and the combination of P-DM and cFaSSIF types.

Tables

**Table S1.** Composition of citrate-phosphate buffer pH 5 used for the preparation of different cFaSSIF types.

| Type | pH [-] | Citrate [mM] | Phosphate [mM] | NaOH [mM] | NaCl [mM] |
| --- | --- | --- | --- | --- | --- |
| Citrate-phosphate buffer | 5 | 36.0 | 36.0 | 114.0 | 84.7 |

**Table S2.** Parameters of in-line analytics using the Cary3500.

| Sample | Type | Wavelength [nm] | Averaging time [s] | Spectral bandwidth [nm] | Scan rate [nm/min] | Cycle [s] |
| --- | --- | --- | --- | --- | --- | --- |
| P-DM | Optical density (OD) | 500 | 0.02 | 1.0 | 1500 | 10 |
| Blank-DM | Optical density (OD) | 500 | 0.02 | 1.0 | 1500 | 10 |

**Table S3.** pH of media in two-stage precipitation assays, donor, and acceptor compartments.

| **Sample** | **pH** |
| --- | --- |
| Blank-DM | 2.99 ±0.02 |
| P-DM | 3.31 ±0.03 |
| P-DM/cFaSSIF_2x_ | 4.07 ±0.00 |
| P-DM/cFaSSIF_10x_ | 3.95 ±0.01 |
| P-DM/cFaSSIF_20x_ | 3.94 ±0.01 |
| SDD-DM | 3.30 ±0.01 |
| SDD-DM/cFaSSIF_2x_ | 4.01 ±0.00 |
| SDD-DM/cFaSSIF_10x_ | 3.87 ±0.01 |
| SDD-DM/cFaSSIF_20x_ | 3.86 ±0.01 |
